# Large‐Scale Synthesis of High‐Purity Isoguanosine and Resolution of its Crystal Structure by Microcrystal Electron Diffraction

**DOI:** 10.1002/open.202400141

**Published:** 2024-06-17

**Authors:** Kaichao Wang, Tiannan Liu, Hang Zhao, Jiang Liu

**Affiliations:** ^1^ State Key Laboratory of Oral Diseases National Center for Stomatology National Clinical Research Center for Oral Diseases Med-X Center for Materials West China Hospital of Stomatology Sichuan University 610041 Chengdu Sichuan P. R. China

**Keywords:** Large-scale synthesis, isoG, High purity, Crystal structure, Hydrogen bond

## Abstract

Isoguanosine (isoG) is a natural structural isomer of guanosine (G) with significant potential for applications in ionophores, genetics, gel formation, and cancer therapy. However, the cost of commercially available isoG on a gram scale is relatively high. To date, a detailed method for the large‐scale preparation of high‐purity isoG has not been reported. This study presented a simple and convenient approach for the large‐scale synthesis of isoG through the diazotization of 2,6‐diaminopurine riboside with sodium nitrite and acetic acid at room temperature. Further, this method could synthesize isoG derivatives (2’‐fluoro‐isoguanosine (**1**) and 2’‐deoxy‐isoguanosine (**2**)) from 2,6‐diaminopurine nucleoside derivatives using diazotization. The structural information of natural and modified nucleosides is crucial for the modification and substitution of DNA/RNA. This study obtained the single‐crystal structure of isoG for the first time and analyzed it in detail using microcrystal electron diffraction. The three‐dimensional supramolecular structure of isoG adopted similarly base‐pair motifs from π‐π stacking interaction of diverse layers, intramolecular hydrogen bonding, and distinct hydrogen bonding interactions from sugar residues. This study has contributed to further isoG modification and its applications in medicinal chemistry and materials.

## Graphical abstract

This study presents a simple and convenient method for the large‐scale synthesis of isoguanosine by diazotization. The single crystal structure of isoG was obtained and analyzed by microcrystal electron diffraction for the first time. The results contribute to the further modification of isoG and its potential applications in medicinal chemistry and materials.

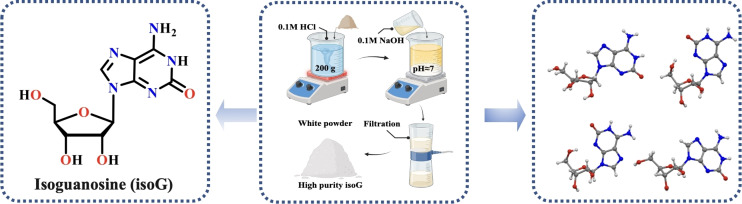

## Introduction


**Figure 1:**
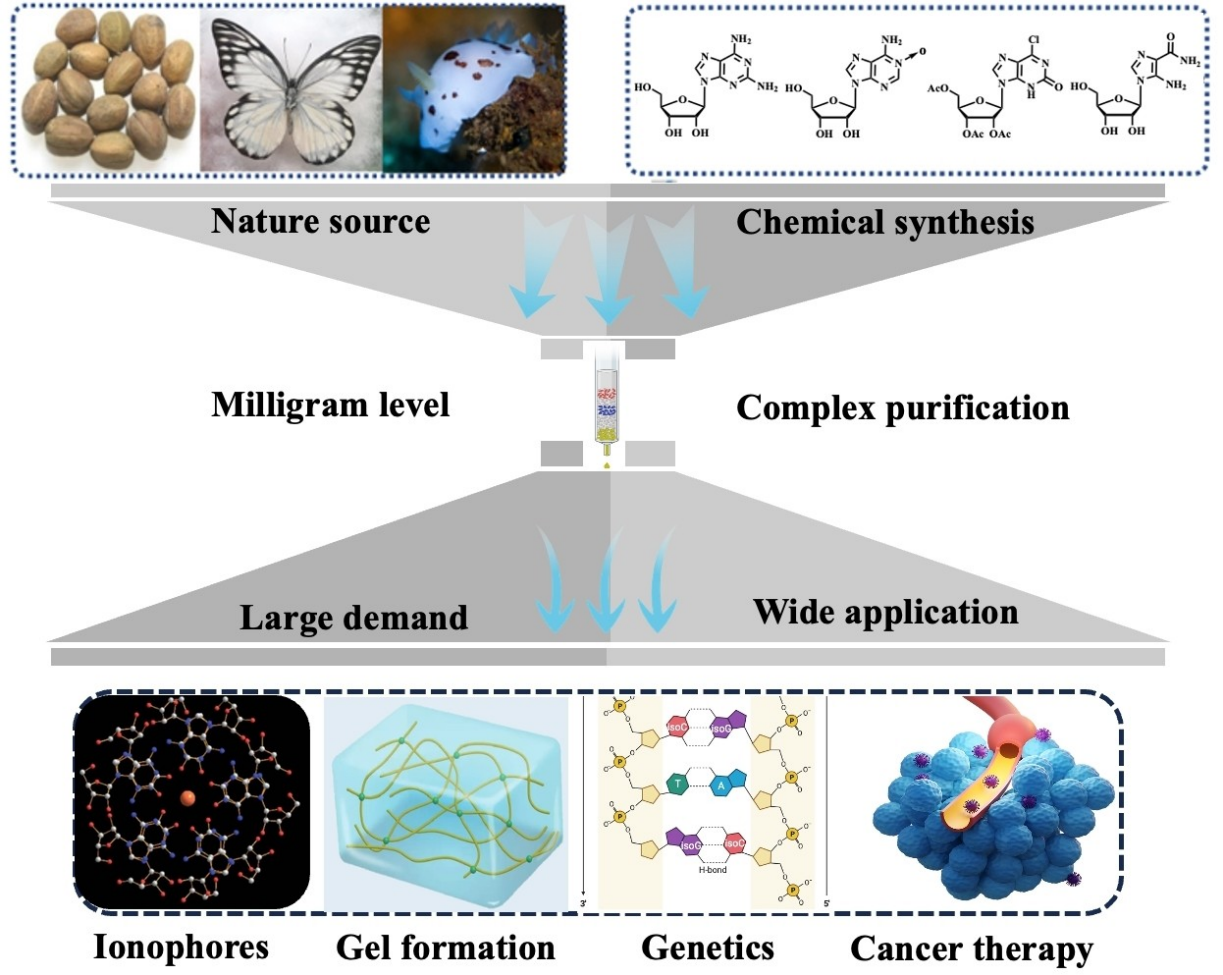
The origin and application of isoG.

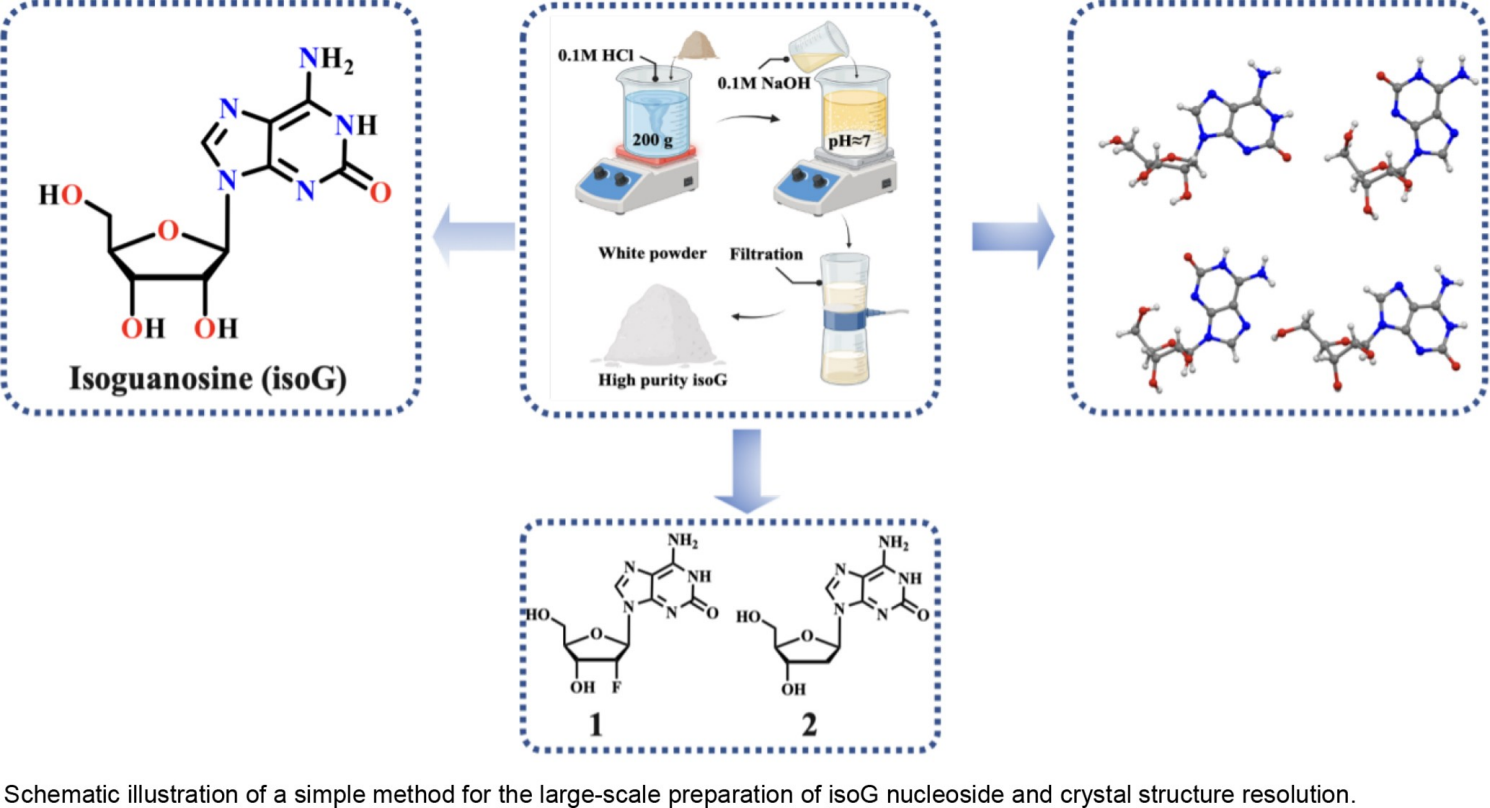
Isoguanosine (isoG), an isomer of guanosine (G), has amino and carbonyl groups transposed relative to G. Cherbulize and Bernhard^[1]^ originally isolated isoG from croton beans, and Davoll^[2]^ subsequently synthesized it. It is widely applied in ionophores, genetics, gel formation, and cancer therapy based on its unique structure and base‐pairing pattern (Figure 1). For example, alkali and alkaline earth ions can self‐assemble the derivatives of G to give the hydrogen‐bonded G quartet.^[3]^ The isoG derivatives self‐associated with high affinity in the presence of Cs^+^ to form isoG_5_ over other alkali and alkali earth metal ions,^[4]^ with the potential to effectively remove cesium‐137.^[5]^ Seela *et al*.^[6]^ reported that the non‐standard 2’‐deoxy‐isoguanosine(isoG_d_)–2’‐deoxycytidine(_d_C)/2’‐deoxy‐isoguanosine(isoG_d_)–2’‐deoxyisocytidine(isoC_d_) base pair could increase/decrease the stability of duplexes in DNA/RNA.^[7]^ Additionally, this property could contribute to developing non‐standard base pairs to extend gene dictionaries and provide a deeper understanding of translation termination.^[8]^ Moreover, Seela *et al*. reported that isoG could self‐assemble to form hydrogel with excellent long‐term stability of more than 3 months in the presence of alkali metal ions.^[9]^ Interestingly, the authors of this study prepared a series of boric acid derivatives and their mediated G hydrogels, which were systematically investigated in oral squamous cell carcinoma. This discovery provided a favorable foundation for developing novel G and isoG‐based supramolecular hydrogels that integrate localized delivery and anti‐cancer activity for targeted tumor therapy.^[10]^

To date, based on the large potential application of isoG, the authors of this study have successfully designed and synthesized a series of isoG‐based dual‐functional hydrogels^[11]^ used for wound healing^[12]^ and anti‐cancer drug release.^[13]^ However, isoG is obtained from natural products since its high purity and large‐scale preparation are costly (Figure 1).^[14]^ Moreover, the traditional extraction method is usually time‐consuming and requires specialized equipment, complicated sample pre‐processing, and high‐expertise operators. Additionally, little progress has been made in the large‐scale preparation of isoG and its derivatives (Table 1). Therefore, developing a large‐scale method to prepare high‐quality isoG is essential.

**Table 1:** The scale synthesis of isoG.

Year	Author	Initial substrates	Reaction scale	References
1932	Cherbuliez *et al*.	Croton tiglium L	Milligram	[1]
1940	Purrmann	Butterly wings	Milligram	[15]
1951	Davoll	2,6‐Diamino‐9‐β‐d‐glucopyranosylpurine	Milligram	[2]
1981	Fuhrman *et al*.	Marine mollusks	Milligram	[16]
1985	Nair *et al*.	Guanosine	Milligram	[17]
1987	Chern *et al*.	The protected AICA riboside	Milligram	[18]
1999	Jurczyk *et al*.	2,6‐Diaminopurine riboside	Gram	[19]
2010	Chatgilialoglu *et al*.	2,6‐Diaminopurine	Milligram	[20]
2024	Liu *et al*.	2,6‐Diaminopurine riboside	Hectogram	This study

This study reported a simple, fast, and convenient method for the large‐scale preparation of high‐quality isoG through the diazotization of 2,6‐diaminopurine riboside with sodium nitrite (NaNO_2_) and acetic acid (AcOH) at room temperature, followed by the N7‐position protonation and deprotonation. Additionally, this method was used to prepare 2’‐fluoro‐isoguanosine (**1**) and 2’‐deoxy‐isoguanosine (**2**). Finally, the single crystal structures of isoG and G were systematically studied and compared. The results showed that the crystal structures of isoG and G formed different patterns of hydrogen bonding (HB) between the nucleobases and/or sugar residues.

## Results and Discussion

### Large‐Scale Preparation of isoG

**Figure 2:**
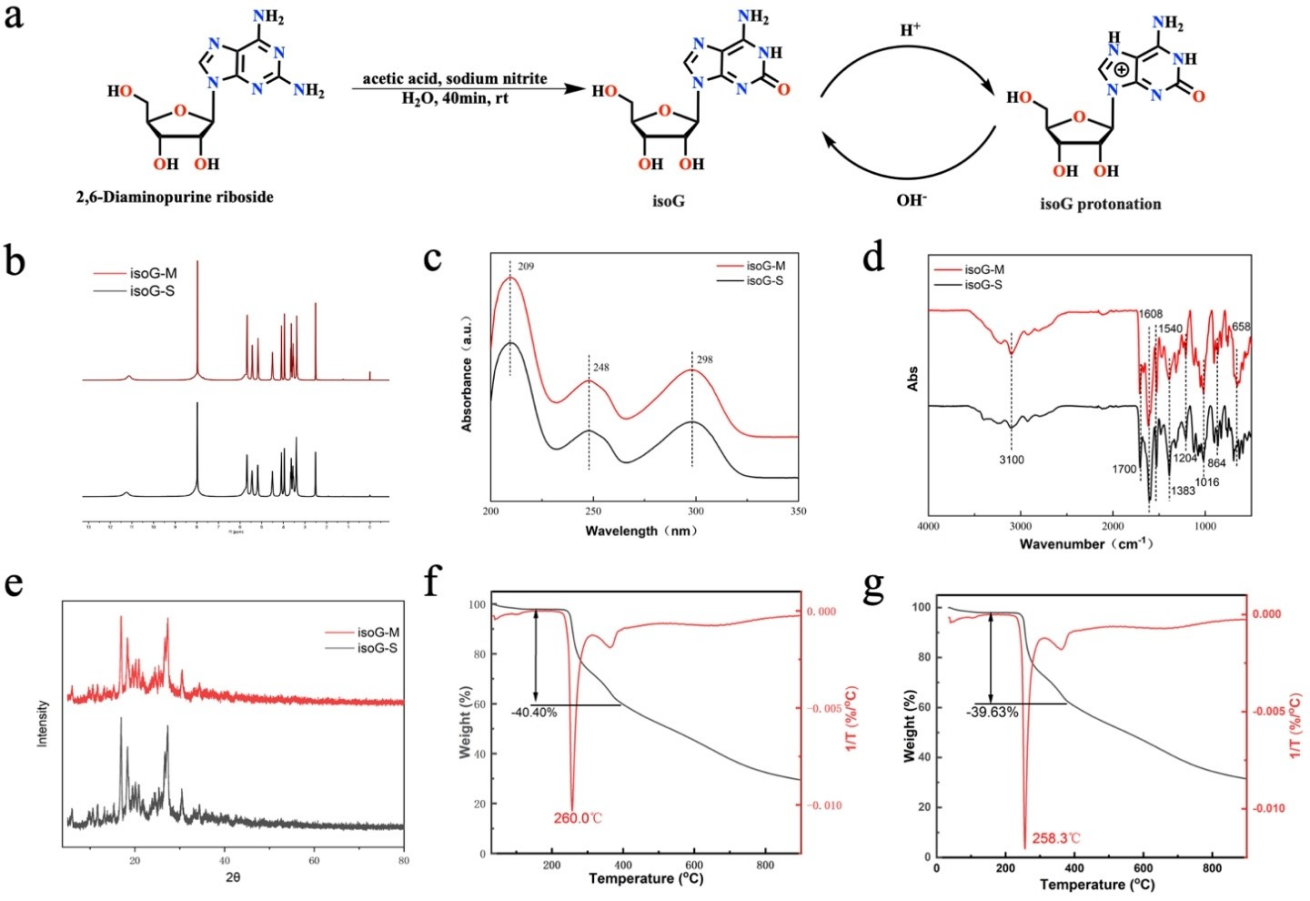
Schematic illustration of large‐scale isoG with high quality prepared and characterization (a) the preparation process of isoG; (b) ^1^H NMR spectra of isoG‐M and isoG‐S; (c) UV spectrum of isoG‐M and isoG‐S; (d) FT‐IR spectra of isoG‐M and isoG‐S; (e) PXRD spectra of isoG‐M and isoG‐S; (f–g) TGA diagrams of isoG‐M and isoG‐S.

**Scheme 1:**
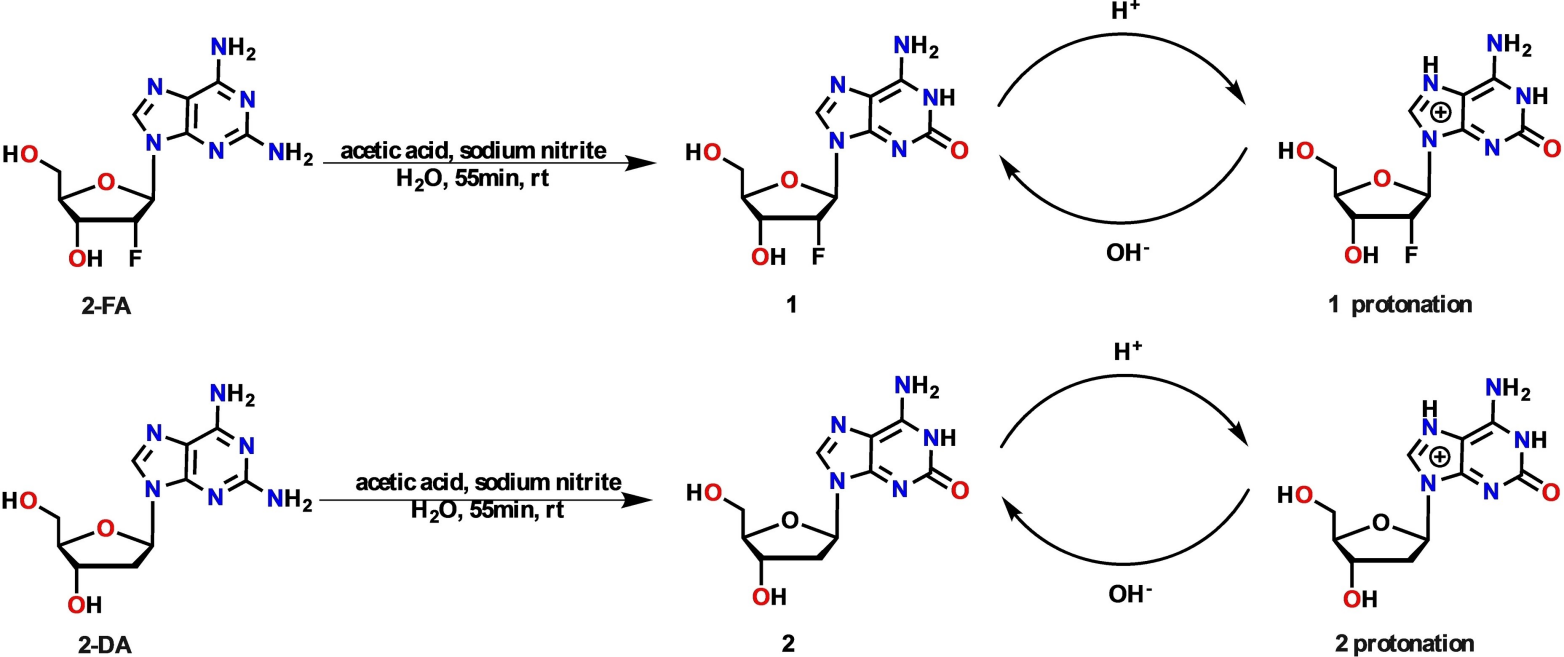
The preparation process of compound **1** and **2**.

The synthesis methods of isoG have been summarized in the previous review. Considering the raw material cost, operation convenience, reaction time and so on, we choose 2,6‐diaminopurine riboside as raw material, followed by treating it with NaNO_2_ and AcOH in water at room temperature.[^19^, ^21^] The reaction finished after 40 min. Then, aqueous ammonia was added to this solution until pH 7 was reached. Then, the crude product was precipitated, which was separated from the mother liquor using filtration. Then, the crude product was treated with 0.1 M hydrochloric acid (HCl) solution for its protonation (N7‐position), and the pH was adjusted to 3 to remove the insoluble impurities. Finally, many high‐quality isoG were deprotonated (N7‐position) and precipitated using 0.1 M sodium hydroxide (NaOH) solution (Figure 2a). Additionally, high‐quality compounds **1** and **2** were successfully synthesized at large‐scale based on these reaction conditions (Scheme 1). The conversion of reactants was determined by ^1^H NMR, utilizing 1,3,5‐trimethoxybenzene as the internal standard. The pre‐treatment yields of the three compounds were determined to be 97.2 % for isoG, 96.0 % for Compound **1**, and 92.4 % for Compound **2**, respectively (Table S1). It is believed that there were three main reasons for the decrease in yield after the purification process. First, in the post‐treatment process, the addition of aqueous ammonia cannot completely precipitate isoG; second, the crude product contained some impurities such as sodium salt and nitrite before purification, and these impurities were removed after purification, resulting in a lower yield; third, during the transfer, filtration and washing process, some products may have appropriate losses, such as small crystals may be washed away through the filter paper pores during filtration. The decrease in yield was not due to deglycosylation.

### Compositional Characterizations of isoG‐M and isoG‐S

The high‐purity isoG prepared in this study and the commercial isoG were named isoG‐M and isoG‐S, respectively. Subsequently, their characteristics were analyzed using proton nuclear magnetic resonance (^1^H NMR), **carbon‐13** nuclear magnetic resonance (^13^C NMR), ultraviolet spectra (UV), fourier transform infrared spectroscopy (FTIR), powder X‐ray diffraction (PXRD), thermogravimetric analysis (TGA), elemental analyzer testing (EA), and high‐performance liquid chromatography (HPLC) (Figure S1–S12). From the ^1^H NMR, the isoG‐M signals at about 10.82 and 8.77–7.17 ppm were ascribed to the protons on the base moiety, the isoG‐M signals at about 3.47–5.87 ppm were attributed to the sugar ring, and these characteristic peaks of isoG‐M were highly consistent with those of isoG‐S (Figure 2b, S5–S6). As seen from the UV spectrum, isoG‐S and isoG‐M had identical UV characteristic peaks, and isoG‐M had pretty high purity than isoG‐S (Figure 2c). Additionally, the FT‐IR spectrum showed that isoG‐M and isoG‐S had almost the same characteristic peaks (Figure 2d). The PXRD analysis of the powdered crystalline solids of isoG‐M and isoG‐S showed that they had quite similar PXRD patterns (Figure 2e). Moreover, the TGA results were consistent with the stability as isoG‐M and isoG‐S lost 40.40 % and 39.63 % weight when heated to 400 °C, respectively (Figure 2f–2 g). Compared to isoG‐C, isoG‐M, and isoG‐S had identical elemental analysis (C, H, and N) (Figure S13). HPLC analysis determined that isoG‐M was 95.97 % and isoG‐S was 95.39 % (Figure S11–S12). The color of the isoG‐M powder and isoG‐S was pure white and light yellow, respectively (Figure S14). Therefore, these results showed the successful large‐scale preparation of high‐quality isoG‐M.

### Single Crystal Structure of isoG

Furthermore, the crystal structure of isoG would furnish additional information about nucleoside conformation, HB capabilities, and the forces governing the stacking of purine rings in the solid state. The detailed structural information of isoG is significantly important to fully understand its biological function. In 2000, Davis *et al*. reported the first X‐ray crystal structure of the 5’‐tert‐butyldimethylsilyl‐2’,3’‐*O*‐isopropylidene‐substituted isoG, providing evidence for the formation of the H‐bonded isoG_5_ (isoG‐star) self‐assembly in the presence of cesium ions.^[4]^

**Figure 3:**
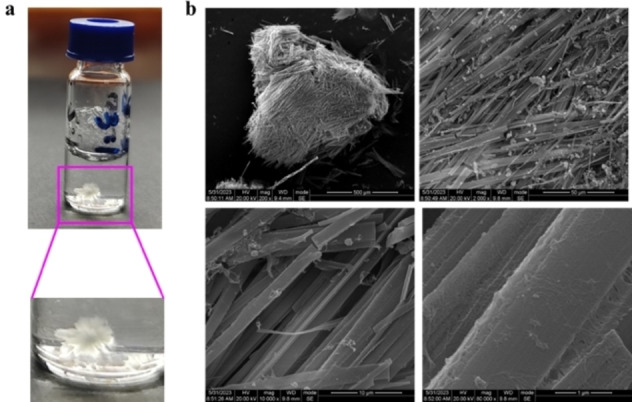
(a) Photographs and (b) scanning electron microscopy (SEM) images of isoG single crystals. Scale bars: 500, 50, 10, and 1 μm.

This study presented a simple and convenient approach for synthesizing isoG on a large scale through diazotization. After several attempts, to our knowledge, the single crystal structure of isoG was obtained for the first time in thermally saturated methanol/H_2_O solution with a volume ratio of 3/1 by a slowly cooling procedure, which lays a foundation for understanding the supramolecular structure of isoG. Microcrystal electron diffraction (MicroED) was used to determine its single‐crystal structure. The single crystals of isoG exhibited a hexagonal snowflake shape (Figure 3a) and looked like dense interwoven fibers (Figure 3b). The crystallographic and refinement information of isoG is shown in Table S2.

**Figure 4:**
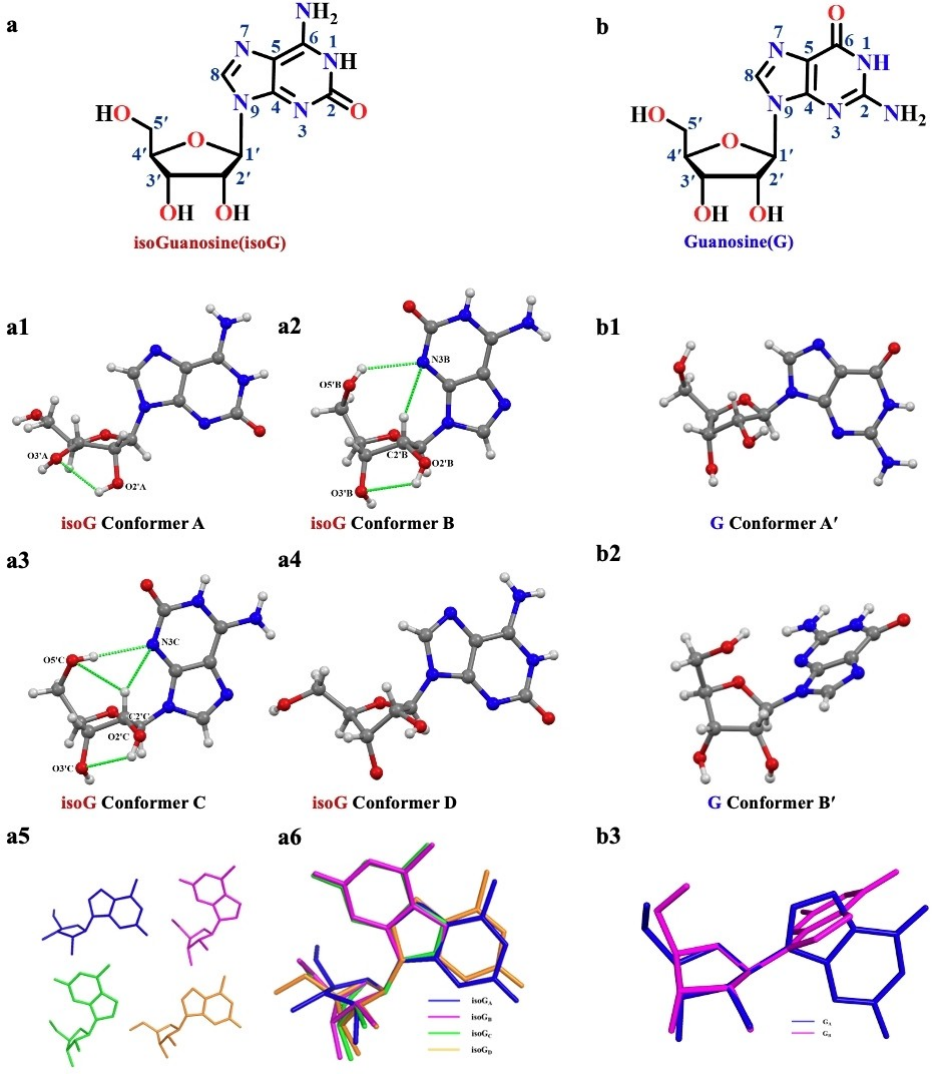
Molecular structures of (a) isoG and (b) G with systematic numbering. (a1–a4) Conformers **i**soG_A_, isoG_B_, isoG_C_, and isoG_D_, respectively. (b1 and b2) Conformers of G. The molecular overlay of isoG A, B, C, and D (a5) and (a6), G _A′_ and _B′_ (b3). Oxygen, nitrogen, carbon, and hydrogen atoms are coded with red, blue, gray, and white, respectively.

The molecular structure and systematic numbering of isoG and G are shown in Figure 4a and 4b. According to the preliminary report published by Bugg *et al*. in 1968,^[22]^ the crystal structures of G ( monoclinic, space group *P*2_1_, cell dimensions of *a*=17.518 Å, *b*=11.502 Å, *c*=6.658 Å, and *β*=98.17°) and its two conformers (G_A’_ and G_B’_) had four water molecules per asymmetric unit (Figure 4 b1–b2). Since the crystals of G were first resolved in 1970 by Walt U, many studies reported the novel function of G and its derivatives based on the unique self‐assembly structure. As mentioned earlier, isoG differs from G in the transposition of the C2 carbon group and the C6 amino group, which is a small change but results in significantly different physical and chemical properties of isoG and G. An apparent characteristic of the single crystal structure of isoG was the existence of four distinct conformers (isoG_A_, isoG_B_, isoG_C_, and isoG_D_). In the canonical pyrimidine and purine nucleosides, the *anti/syn* conformation of the glycosyl bond is vital for related information. In the case of purine nucleosides, the single crystal structure of G had two conformers (G_A′_ and G_B′_), which adopt *anti* (−137.1(7)°) and *syn* (−58.0(7)°) conformations. However, the torsion angle χ (O4’−C1’−N9−C4) could define the orientation of the nucleobase relative to the sugar ring for isoG. The torsion angle χ measured for conformers isoG_A_, isoG_B_, isoG_C_, and isoG_D_ was −135.7(7)°, 57.6(4)°, 57.8(1)°, and −111.6(0)°, implying that conformers isoG_A_ and isoG_D_ adopted *anti* conformation while conformers isoG_B_ and isoG_C_ adopted *syn* conformation.

Additionally, the intramolecular HBs are important for molecular self‐assembly. According to the geometry of HBs of isoG, the differences in these parameters may be attributed to the intramolecular HBs in conformers isoG_A–D_. Except conformer isoG_D_, conformers isoG_A_, isoG_B_, and isoG_C_ had intramolecular HBs (O2’−H−−O3’). Moreover, conformers isoG_B_ and isoG_C_ had two weak HB interactions (C2’−H−−N3, C2’−H−−O5’) and one normal HB interaction (O5’−H−−N3) (Figure 4 a1–a4). Finally, the overlap graphs of isoG and G showed that the molecular shapes of the corresponding conformers of the nucleobase were almost superimposable, and the four conformers of isoG and two conformers of G had primary differences in the sugar (Figure 4 a5, a6, b3, and S15). Therefore, the crystal structure information stated revealed the differences in the chemical and physical properties of isoG and G (Table S3–S6). Pseudorotation phase angle (*P*) and the maximum puckering amplitude (τ_m_) defined sugar puckering, which is the driving force of all conformations of nucleosides. *P* presented in natural and modified nucleosides had two ranges: C3’‐*endo* with 0°≤*P*≤36° (North, *N*‐type) or C2’‐*endo* with 144°≤*P*≤180° (South, *S*‐type). isoG_A_ adopted a nonclassical conformation with a twist of C4’‐*exo* (*S*‐type, *P*=36.8(8)°, τ_m_=28.3(0)°, and ^3^T_2_ twist form). isoG_B_ adopted a typical *N*‐type conformation with a twist of C2’‐*endo* (*S*‐type, *P*=147.1(0)°, τ_m_=40.6(7)°, and ^2^T_3_ sugar puckering model). isoG_C_ adopted C1’‐*exo* (*S*‐type, *P*=137.6(4)°, τ_m_=51.7(7)°, and ^2^T_3_ sugar puckering model) and isoG_D_ adopted C3’‐*exo* (*S*‐type, *P*=182.1(1)°, τ_m_=52.4(4)°, and ^2^T_3_ sugar puckering model). The torsion angle γ (O5’−C5’−C4’−C3’) defined the orientation of the 5’‐hydroxyl group relative to the sugar ring. isoG_A_ and isoG_C_ were in *ap* (*gauche*, *trans*) range, isoG_B_ was in +*sc* (*gauche*, *gauche*) range, and isoG_D_ was in −*sc (trans*, *gauche*) range.

### The Solid‐State Intermolecular Interaction of isoG

**Figure 5:**
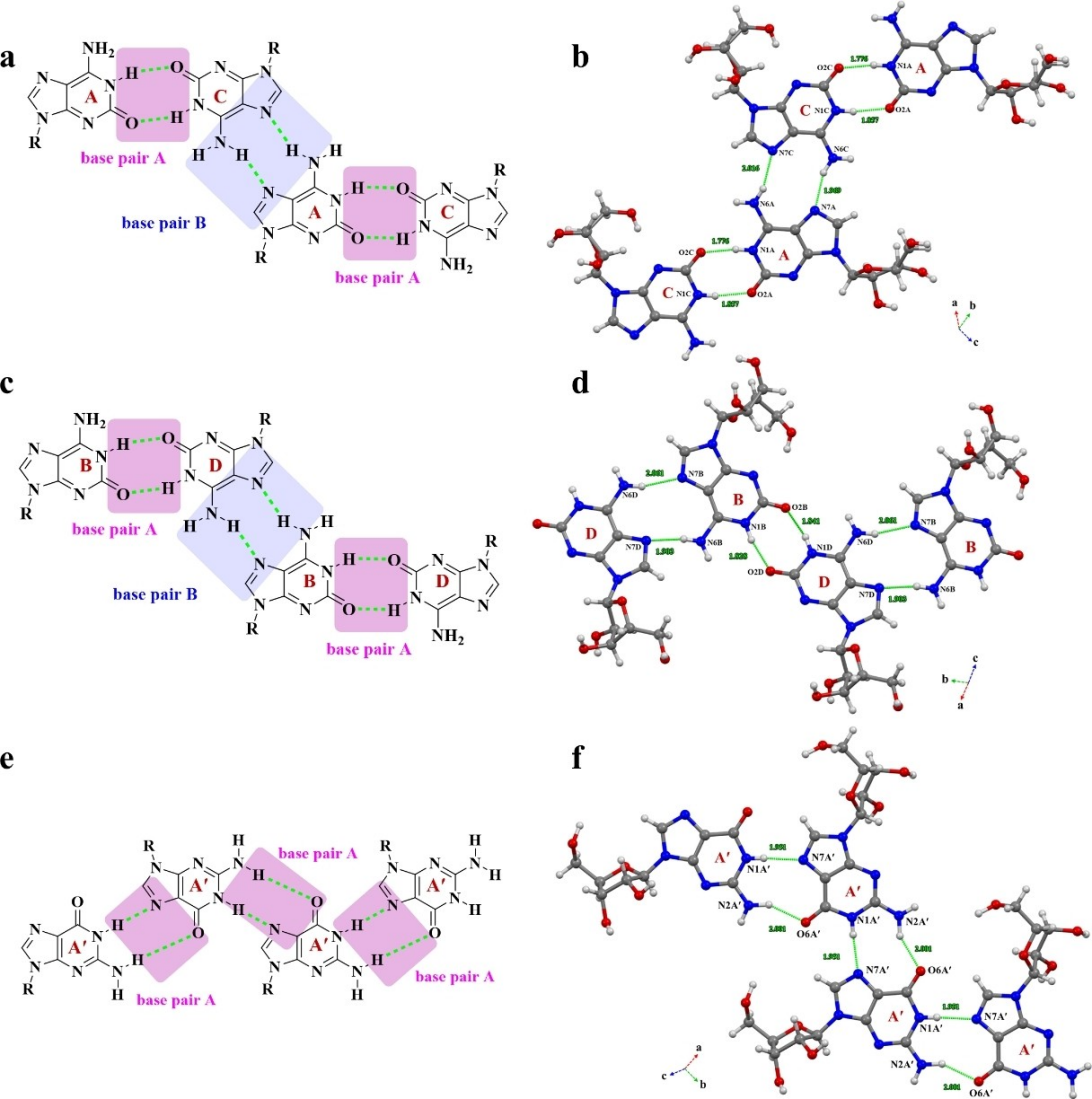
The base‐pair pattern of isoG and G. The base‐pair pattern of conformer isoG_A_ and isoG_C_ a)–b), conformer isoG_B_ and isoG_D_ c)–d). The base‐pair pattern of conformer G_A′_ and G_A′_ or G_B′_ and G_B′_ e)–f). Atoms are coded as follows: red, oxygen; blue, nitrogen; gray, carbon; white, hydrogen.

The complex hydrogen networks of isoG consisted of three categories at the three‐dimensional (3D) level: base‐base, sugar‐sugar, and base‐sugar interactions from multiple layers with subtle distinctions. First, the base‐pair patterns of isoG were systematically investigated and compared with the G nucleoside. As mentioned before, the single crystal structure of isoG had four conformers. The intermolecular HBs (N1−H−−O2, N6−H−−N7, d_N1−O2_=2.722 Å, d_N6A−N7C_=2.862 Å, and d_N6C−N7A_=2.812 Å) mediated the self‐association of isoG for conformers isoG_A_ and isoG_C_ (Figure 5a–5b). Additionally, the intermolecular HBs (N1−H−−O2, N6−H−−N7 d_N1−O2_=2.692 Å, and d_N6−N7_=2.772 Å) mediated the self‐association of isoG for conformers isoG_B_ and isoG_D_ (Figure 5c–5d). The base pairs of conformers isoG_A_ and isoG_C_ had a bending angle of 15.81°, and conformers isoG_B_ and isoG_D_ had a bending angle of 13.92° (Figure S16). However, the base‐pair patterns for G are joined by two HBs (N1−H−−N7 and N2−H−−O6) from two conformers, and these two HBs had different bond lengths compared to isoG (d_N1A−N7A_=2.817 Å, d_N1B−N7B_=2.876 Å, d_N2A−O6A_=2.918 Å, and d_N2B−O6B_=2.990 Å) (Figure 5e–5f). The structure featured hydrogen bonding between purine bases to form ribbons parallel to b and parallel stacking of purine bases along c. The separation between adjacent rings within a stack was 3.3 Å. The conformations about the glycosidic C−N bond and the puckerings of the sugar rings were quite different for the two molecules in the asymmetric unit.

**Figure 6:**
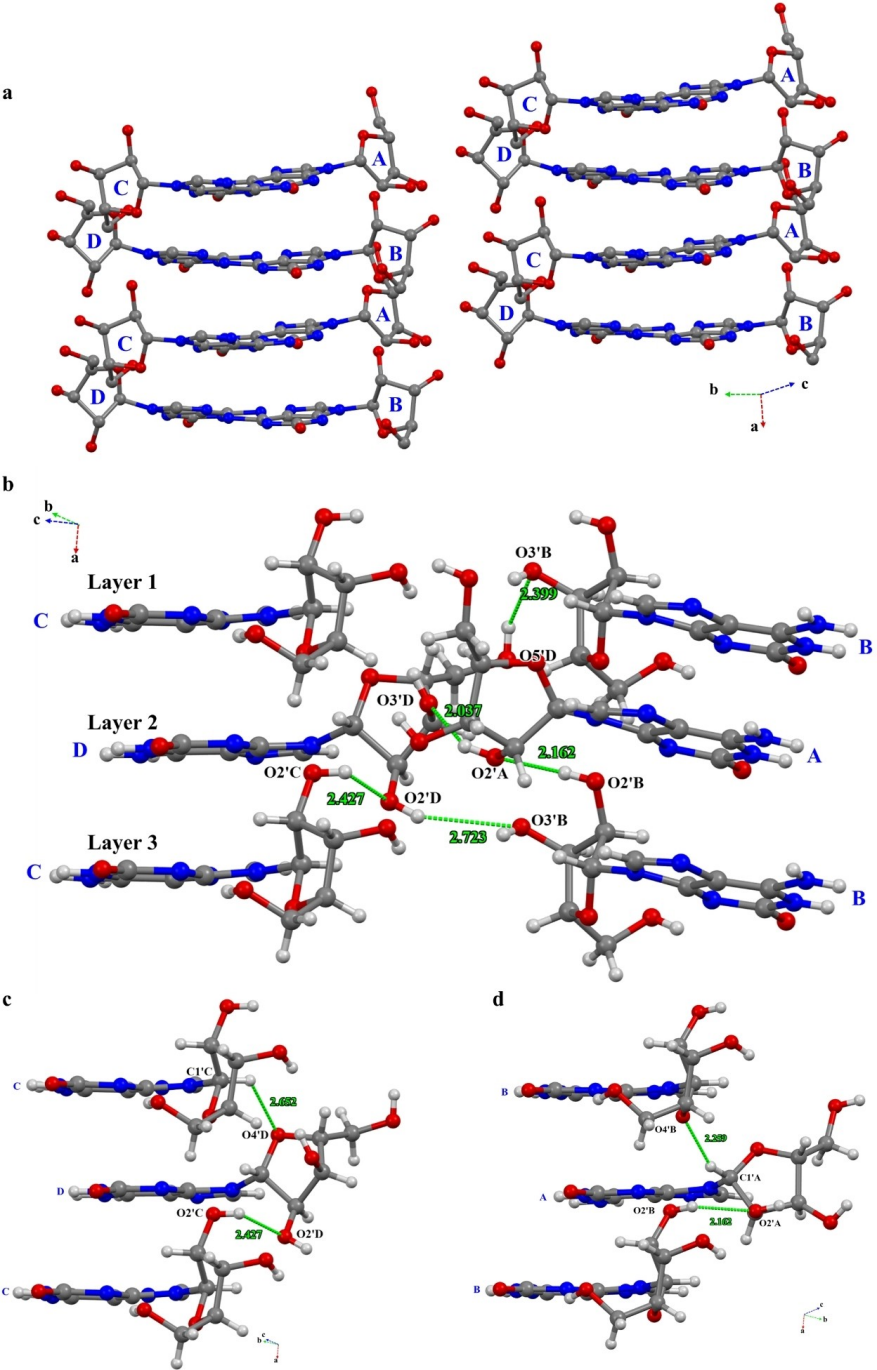
Complicated hydrogen bond networks of isoG between sugar and sugar. a) The overall packing mode of isoG. b), c) and d) The layer–layer interactions based on the HBs between sugars. Atoms are coded as follows: red, oxygen; blue, nitrogen; gray, carbon; white, hydrogen.

Furthermore, conformers isoG_A_ to isoG_D_ of isoG self‐assembled into a regular 3D structure with the hydrophobic nucleobases stacked into layered ribbons by specific spatial and interaction modes (Figure 6a), and the hydrophilic sugar residues associated with the backbones. The authors of this study had already analyzed the HB interaction of the nucleobase to nucleobase. Additionally, the sugar‐to‐base interaction was crucial for the 3D structure. The crystal structure of isoG had two weak HBs from conformer isoG_B_ to conformer isoG_A_ (C8_B_−H8_B_−−O4’_A_) and conformer isoG_C_ to conformer isoG_D_ (C8_C_−H−−O4’_D_), which contributed to the stability of the 3D structure of the adjacent layers.

Finally, strong and weak HBs from the sugar to sugar were other interactions of the hydrophilic backbone (Figure 6a). Conformer isoG_B_ had two strong HBs from layers 2 and 3, where one was formed by conformers isoG_D_ and isoG_B_ (O2’_D_−H−−O3’_B_) and the other one was formed by conformers isoG_B_ and isoG_A_ (O2’_B_−H−−O2’_A_) (Figure 6b). Additionally, conformers isoG_C_ and isoG_D_ formed a strong HB (O2’_C_−H−−O2’_D_) between layers 2 and 3, and the above 3 strong HB made a much more tight hydrophilic backbone. Moreover, another strong HB from conformer isoG_A_ to isoG_D_ (O2’_A_−H−−O3’_D_) also favored the stability of the hydrophilic backbone in layer 2. Besides, another typical HB conformer isoG_D_ to isoG_B_ (O5’_D_−H−−O3’_B_) promoted the robustness of the hydrophilic backbone. In the end, two weak HBs (Conformers isoG_A_ to isoG_B_, C1’_A_−H−−O4’_B_ and conformers isoG_C_ to isoG_D_, C1’_C_−H−−O4’_D_) were taken as an example (Figure 6c–6d), and they were also present between layers 1 and 2 with another non‐conventional HBs (C−H−−O/N) (Figure S17–S25), which was also important for crystal packing.

**Figure 7:**
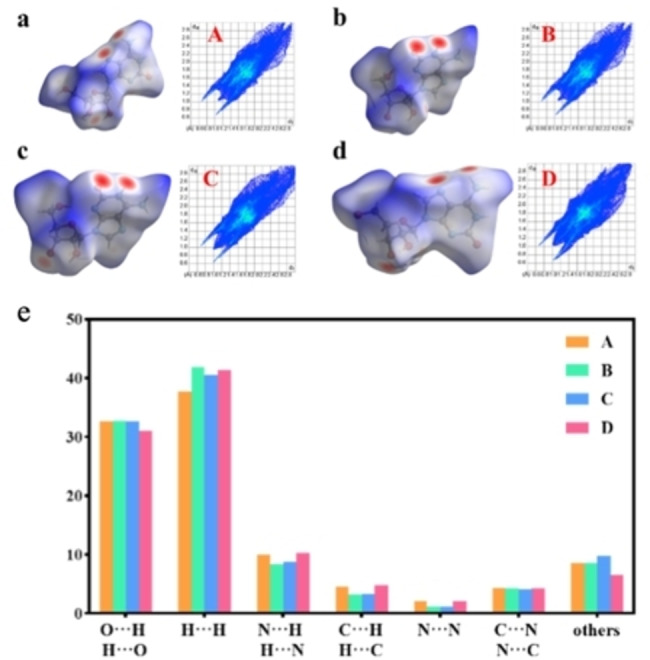
The 2D‐FP and HSA (d_norm_) of (a) isoG_A_, (b) isoG_B_, (c) isoG_C_, and (d) isoG_D_. (e) The individual atomic contact percentages of isoG_A–D_. Oxygen, nitrogen, carbon, and hydrogen atoms are coded with red, blue, gray, and white, respectively.

Furthermore, the Hirshfeld surface analysis (HSA) and associated two‐dimensional fingerprint plots (2D‐FP) were used to reveal more intuitive and clearer intermolecular interactions of the four conformers in the isoG crystal structure. The surfaces were mapped using their d_*norm*_, shape index, and curvedness (Figure 7 and Figure S26–S29). In Hirshfeld surfaces images, white dots represented forces equivalent to the interatomic distances of the van der Waals forces, blue represented the weak forces longer than van der Waals forces, and red represented the distances shorter than van der Waals forces.^[23]^ The distinctive bright red dots in the HSA maps represented sites of the formation of strong HBs, such as H−−O, O−−H, N−−H, and H−−N, while most of the blue regions belonged to weaker interactions and π−π stacking, such as extensive H−H or typical N−N and C−N stacking interactions (Figure 7a–7d).^[24]^ In Figure 7a–7d, a1 reprented N6 as a HB donor (N6_A_−H6_AA_−−N7_C_) and a2 reprented N7 as a HB acceptor (N6_C_−H6_CB_−−N7_A_). Similarly, b1, c1, and d1 reprented N1, N1, and N6 as HB donors, respectively. b2, c2, and d2 reprented O2, O2, and N7 as HB acceptors, respectively. Furthermore, this HB information corresponds to the information listed in Table S7. The red dots at the edges of each surface indicated that π‐π stacking supported the layer. Additionally, it could be found that H−−H, O−−H, and N−−H were the three primary intermolecular interactions (Figure 7e). H−−H interaction was distributed in the middle region of the fingerprints and was the most dominant mode of interaction, with the largest contribution of 41.9 % to the Hirshfeld surface. O−−H with a contribution ratio of 32.7 %, corresponded to O−H−−N, O−H−−O, and N−H−−O. This indicated that the sugar‐ring interactions and base‐pairing also contributed significantly to the self‐assembly of isoG. The π‐π stacking had a larger contribution to the overall assembly for higher proportions of H−H and N−N interactions, which was consistent with the self‐assembly structure of isoG where interlayer intermolecular hydrogen bonding supported π‐π stacking interactions between layers.^[25]^

## Conclusions

In summary, a simple and convenient method was constructed for the large‐scale preparation of isoG through the diazotization of 2,6‐diaminopurine riboside with NaNO_2_ and AcOH at room temperature, which could obtain high‐purity isoG through N7‐position protonation and deprotonation. First, the crude product was treated with 0.1 M HCl solution for protonation and the pH was adjusted to 3 to remove the insoluble impurities. Second, several high‐quality isoG were deprotonated and precipitated through the association with 0.1 M NaOH solution. According to the authors of this study, this was the first time that the single crystal structure of isoG was obtained and resolved using MicroED. Like the G nucleosides, isoG could form ribbon‐like base pairs (conformers isoG_A–D_: N1−H−−O2, N6−H−−N7) without ion template in the solid state. Additionally, the four conformers in isoG adopted the different back‐bond HB connectivity between the base and sugar moieties modes. This study focused on the structural information and large‐scale preparation of isoG, representing a significant potential for its widespread application. The authors of this study intend to study the derivatives of isoG within different functionalized groups in the future for materials and medicinal chemistry.

## Experimental Section

### General Remarks

All the chemicals used were commercially available. Analytical grade solvents and reagents were used without further purification. Thin‐layer chromatography (TLC) was performed on an aluminum sheet covered with silica gel (0.2 mm, 60 F254, Merck, Germany). NMR spectra were recorded on a 400 Hz spectrometer (Bruker ADVANCE) in DMSO‐*d*_6_, and the δ values in ppm were relative to Me_4_Si as the internal standard. Quantitative NMR was performed using 1,3,5‐trimethoxybenzene as the internal standard and DMSO‐*d_6_* as the solvent. High‐resolution mass spectra were measured with a mass analyzer (Q‐TOF, Bruker, Germany). The UV spectra were recorded on a spectrophotometer (DU‐800, Beckman, US), with λ_max_ in nm and ϵ in dm^3^ mol^−1^ cm^−1^. PXRD experiments were performed using a diffractometer (Panalytical Empyrean) equipped with a hybrid monochromator for Cu*K*_α1_ radiation and a detector (PIXcel3D). The samples were analyzed using TGA and differential scanning calorimetry (DSC) on a thermal analyzer (Universal V2.5H). The operating conditions for HPLC (Shimadzu, Japan): A Diamonsil C‐18 column (4.6 mm×250 mm, 5 μm) was used, the flow rate was 1 mL/min, and the column temperature was 25 °C. The mobile phase consisted of methanol (100 %), and the detection wave‐length was 254 nm. The reagent water used was pretreated with the Milli‐Q Plus System.

### Synthesis of isoG and Compounds 1 and 2

**Procedure for synthesizing isoG**. First, 200 g of 2,6‐diaminopurine riboside (0.71 mol) was suspended in 4 L of H_2_O at room temperature. Then, 1 L of AcOH (17.4 mol) was added over 5 min. Then, 122 g of NaNO_2_ (1.76 mol) in 1 L of H_2_O was added dropwise. The clear resultant solution was stirred for 40 min to obtain a yellow solution, whose pH was adjusted to 7 with 2.8 % aqueous NH_3_ in an ice water bath. The obtained precipitate was dissolved in 0.1 M HCl using heat, and active charcoal was added. After hot filtration, the filtrate was cooled in an ice bath and neutralized with 0.1 M NaOH. Then, filtration was used to remove the solvent. Finally, the solid was washed with ice water and vacuum‐dried to yield a white powder. Yield: 82 g (41 %). ^1^H NMR (400 MHz, DMSO‐*d*_6_), δ 10.81 (s, 1H, NH), 7.95 (s, 2H, NH_2_), 7.68 (s, 1H, C8−H), 5.65 (d, *J*=6.4 Hz, 2H, C1’−H and C2’−OH), 5.39 (d, *J*=6.1 Hz, 1H, C3’−OH), 5.12 (d, *J*=3.5 Hz, 1H, C5’−OH), 4.59–4.43 (m, 1H, C2’−H), 4.07 (q, *J*=4.3 Hz, 1H, C3’−H), 3.93 (d, *J*=2.6 Hz, 1H, C4’−H), 3.63 (dd, 1H, C5’−H), 3.52 (d, *J*=11.0 Hz, 1H, C5’−H); ^13^C NMR (101 MHz, DMSO‐*d*_6_) δ 156.35 (1 C, C2), 152.28 (1 C, C6), 138.79 (1 C, C4), 132.34 (1 C, C8), 110.07 (1 C, C5), 88.23 (1 C, C1’), 86.50 (1 C, C4’), 73.52 (1 C, C2’), 71.28 (1 C, C3’), 62.20 (1 C, C5’); HRMS (ESI‐TOF) m/z calcd for C_10_H_13_N_5_O_5_ [M+H]^+^ 284.0995, found 284.0985.

**Procedure for the synthesis of compound 1**. First, 200 g of 2‐amino‐2’‐deoxy‐2’‐fluoro‐d‐adenosine (2‐FA) (0.70 mol) was suspended in 4 L of H_2_O at room temperature. Then, 0.99 L of AcOH (17.2 mol) was added over 5 min. Then, 118 g of NaNO_2_ (1.74 mol) in 1 L of H_2_O was added dropwise. The clear resultant solution was stirred for 55 min to obtain a yellow solution, whose pH was adjusted to 7 with 2.8 % aqueous NH_3_ in an ice water bath. The obtained precipitation was dissolved in 0.1 M HCl using heat, and active charcoal was added. After hot filtration, the filtrate was cooled in an ice bath and neutralized with 0.1 M NaOH. Then, filtration was used to remove the solvent. Finally, the solid was washed with ice water and vacuum‐dried to yield a light yellow powder. Yield: 86 g (43 %). ^1^H NMR (400 MHz, DMSO‐*d*_6_) δ 10.75 (s, 1H, NH), 8.00 (s, 1H, C8−H), 6.01 (dd, *J*=16.2, 3.4 Hz, 1H, C1’−H), 5.71 (d, *J*=5.6 Hz, 1H, OH), 5.33 (dt, *J*=53.0, 3.9 Hz, 1H, C2’−H), 4.44–4.33 (m, 1H, C3’−H), 3.95 (dt, *J*=4.4, 2.0 Hz, 1H, C4’−H), 3.71 (dd, *J*=12.5, 2.6 Hz, 1H, C5’−H), 3.57 (dd, *J*=12.4, 3.6 Hz, 1H, C5’−H); ^13^C NMR (101 MHz, DMSO‐*d*_6_) δ 156.18 (1 C, 2 C), 152.42 (1 C, C6), 138.06 (1 C, C4), 132.27 (1 C, C8), 109.62 (1 C, C5), 93.41 (d, *J*=186 Hz, 1 C, C2’), 85.73 (d, *J*=32 Hz, 1 C, C1’), 84.83 (1 C, C4’), 68.82 (d, *J*=16 Hz, 1 C, C3’), 60.97 (1 C, C5’); HRMS (ESI‐TOF) m/z calcd for C_10_H_12_FN_5_O_4_ [M+H]^+^ 286.0952, found 286.0942.

**Procedure for the synthesis of compound 2**. First, 200 g of diaminopurine 2’‐deoxyriboside(2‐DA) (0.75 mol) was suspended in 4 L of H_2_O at room temperature. Then, 1.06 L of AcOH (18.4 mol) was added over 5 min. Then, 128 g of NaNO_2_ (1.86 mol) in 1 L of H_2_O was added dropwise. The clear resultant solution was stirred for 55 min to obtain a yellow solution, whose pH was adjusted to 7 with a 2.8 % aqueous NH_3_ in ice water bath. The obtained precipitation was dissolved in 0.1 M HCl using heat, and active charcoal was added. After hot filtration, the filtrate was cooled in an ice bath and neutralized with 0.1 M NaOH. Then, filtration was used to remove the solvent. Finally, the solid was washed with ice water and vacuum‐dried to yield a white powder. Yield: 89 g (44.5 %). HRMS (ESI‐TOF) m/z calcd for C_10_H_13_N_5_O_4_ [M+H+H_2_O]^+^ 286.1151, found 286.0943.

### Single Crystal Structure Elucidation of isoG

isoG was dissolved completely in the system with a 3 : 1 ratio of methanol and water by heating it to 80 °C. Consequently, the isoG single crystal was obtained using gradient cooling (80 to 65 °C to finally reach 25 °C). Finally, the single‐crystal structure of isoG was elucidated using MicroED.

Concretely speaking, the collection of electron diffraction uses a transmission electron microscope (JEM‐2100 Plus, Japan Electronics Corporation), which uses a 200 kV accelerating volta ge and is equipped with a high‐speed direct electron camera (MerelinEM). QUANTIFOILgrids (R1.2/1.3), Fischone 2550 Cryo Transfer Tomography Holder, Temperature: 77 K. Sample preparation method: (1) An appropriate amount of eutectic sample was placed in the copper mesh, which was placed in a centrifuge tube with gentle shaking to help the sample stick to the copper mesh. (2) The copper mesh was loaded into the sample rod, which was inserted into the sample compartment of the lens barrel. Then liquid nitrogen was added to the dewar bottle, waiting for the temperature to drop to 100 K. The sample was ready for the test after the vacuum level reached the working state.

Deposition Numbers 2326604 (for isoguanosine) contain the supplementary crystallographic data for this paper. These data are provided free of charge by the joint Cambridge Crystallographic Data Centre and Fachinformationszentrum Karlsruhe Access Structures service.

## Conflict of Interests

The authors declare no conflict of interest.

1

## Supporting information

As a service to our authors and readers, this journal provides supporting information supplied by the authors. Such materials are peer reviewed and may be re‐organized for online delivery, but are not copy‐edited or typeset. Technical support issues arising from supporting information (other than missing files) should be addressed to the authors.

Supporting Information

## Data Availability

The data that support the findings of this study are available from the corresponding author upon reasonable request.
