# Supplementary material for: Large‐Scale Synthesis of High‐Purity Isoguanosine and Resolution of its Crystal Structure by Microcrystal Electron Diffraction
Source: ChemistryOpen. 2024 Jun 17;13(10):e202400141. doi: 10.1002/open.202400141 (PMC12056916; doi:10.1002/open.202400141)
Supplement: Supplementary file 1 — Supporting Information [file OPEN-13-e202400141-s001.pdf]

ChemistryOpen

Supporting Information

Large-Scale Synthesis of High-Purity Isoguanosine and Resolution of its Crystal Structure by Microcrystal Electron Diffraction

Kaichao Wang, Tiannan Liu, Hang Zhao, and Jiang Liu*

Supporting Information

Large-Scale Synthesis of Isoguanosine Nucleoside and Resolution of its Crystal Structure by Microcrystal Electron Diffraction

Kaichao Wang+[a], Tiannan Liu+[a], Hang Zhao[a] and Jiang Liu*[a],

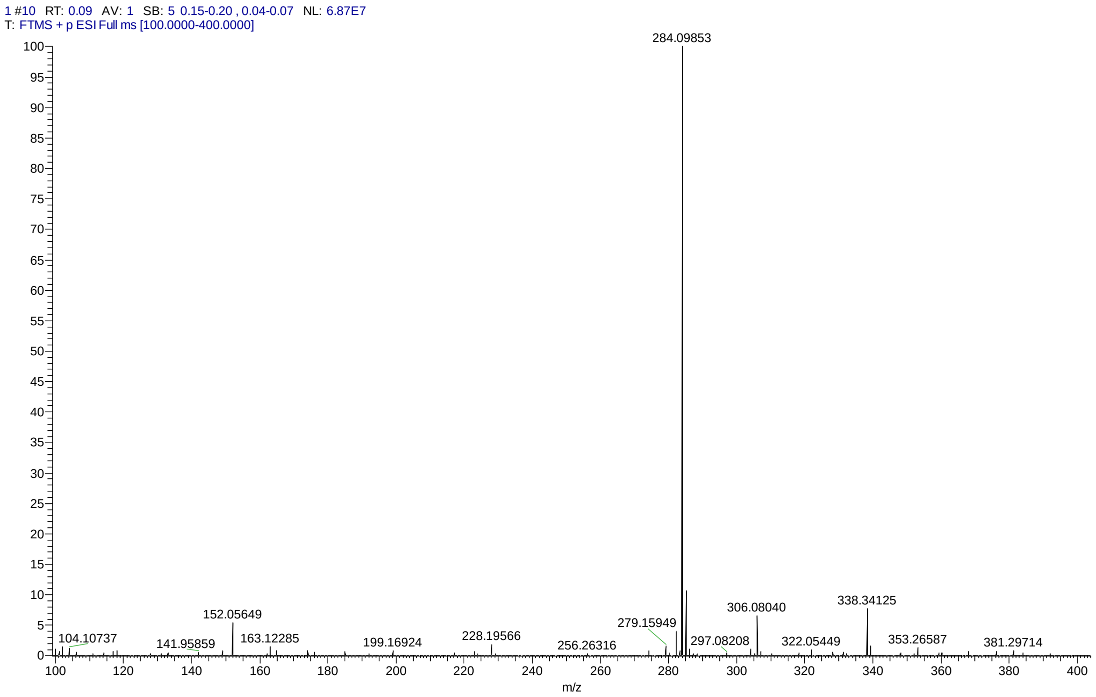

Figure S1. HRMS data of compound isoG-M

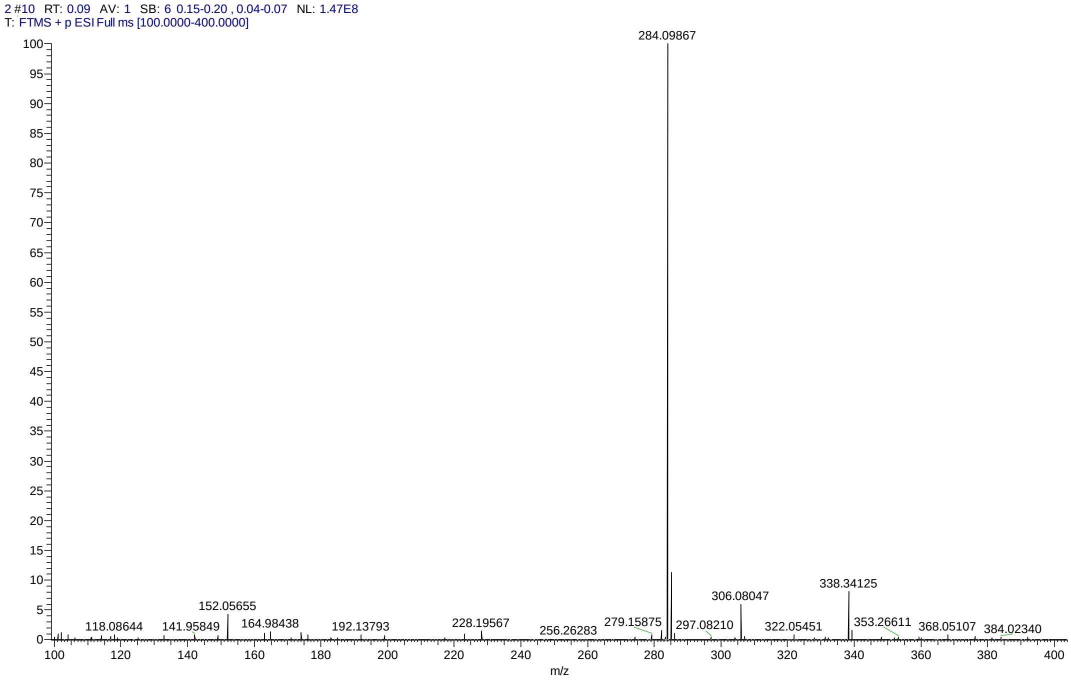

Figure S2. HRMS data of compound isoG-S

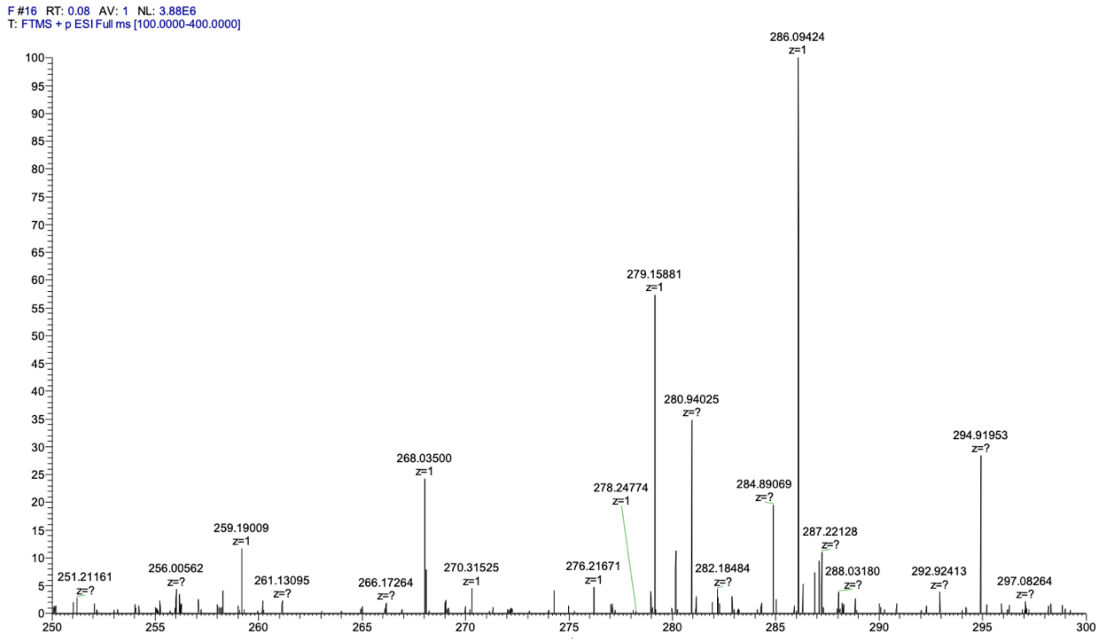

Figure S3. HRMS data of 2’-fluoro-isoguanosine

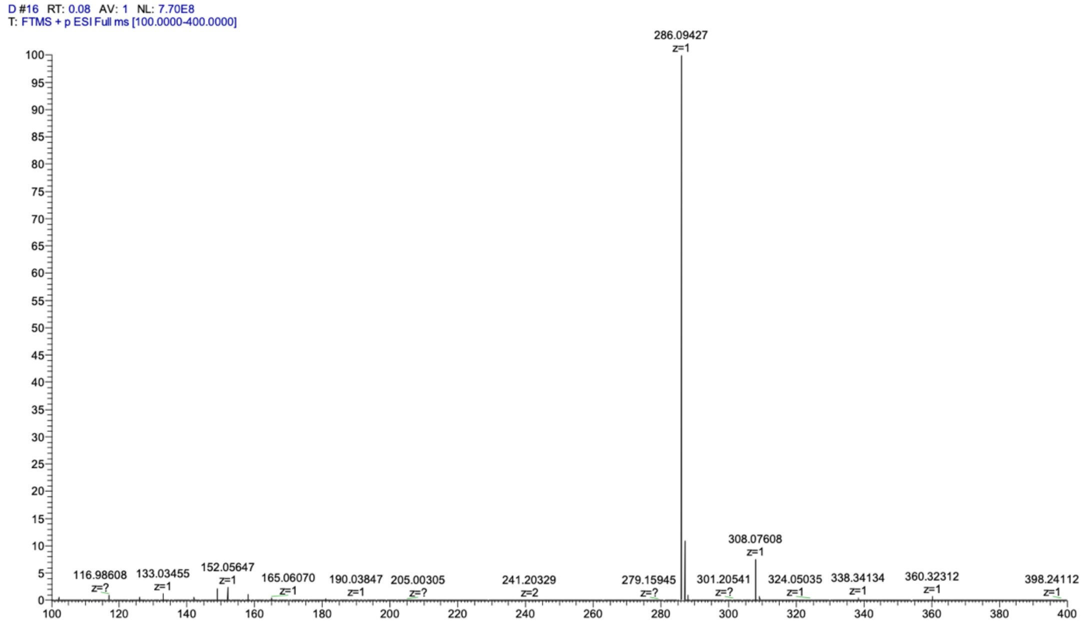

Figure S4. HRMS data of compound 2’-deoxy-isoguanosine

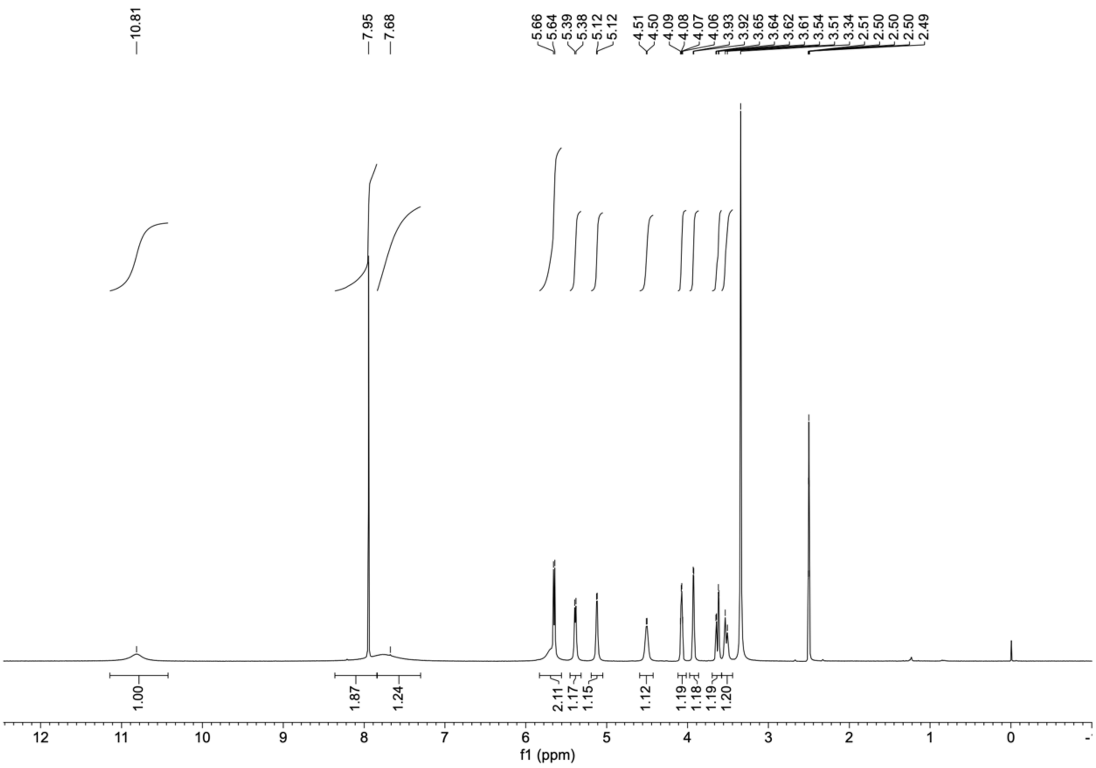

Figure S5. ^{1}H NMR spectrum of compound isoG-M

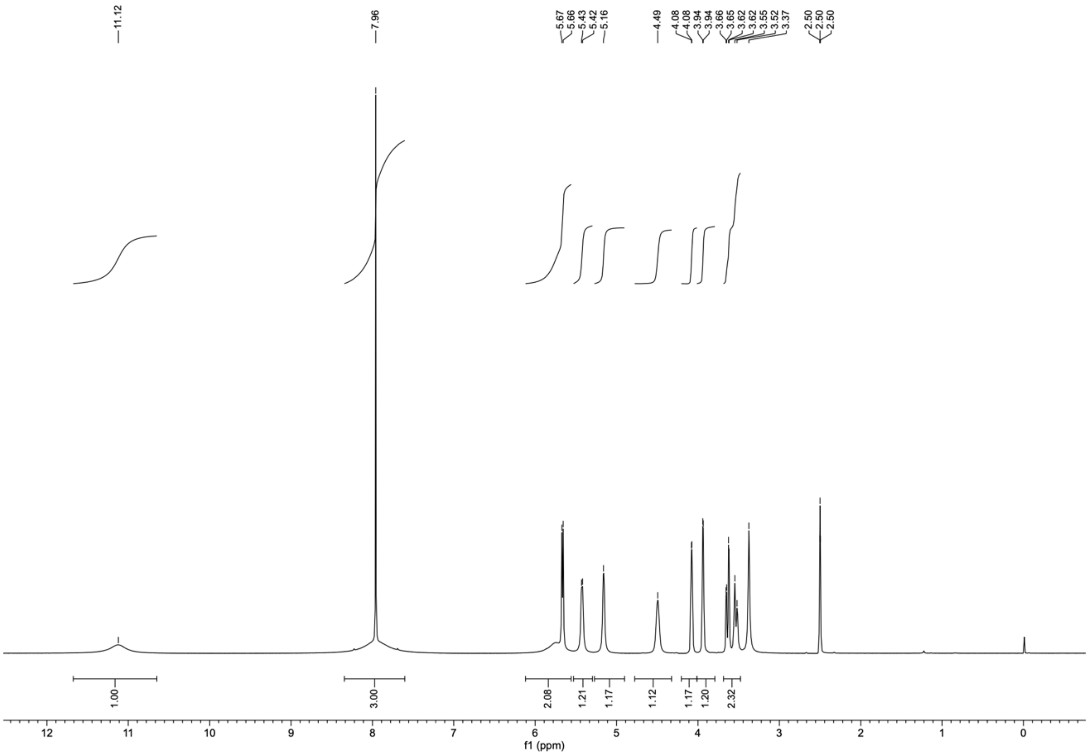

Figure S6. ^{1}H NMR spectrum of compound isoG-S

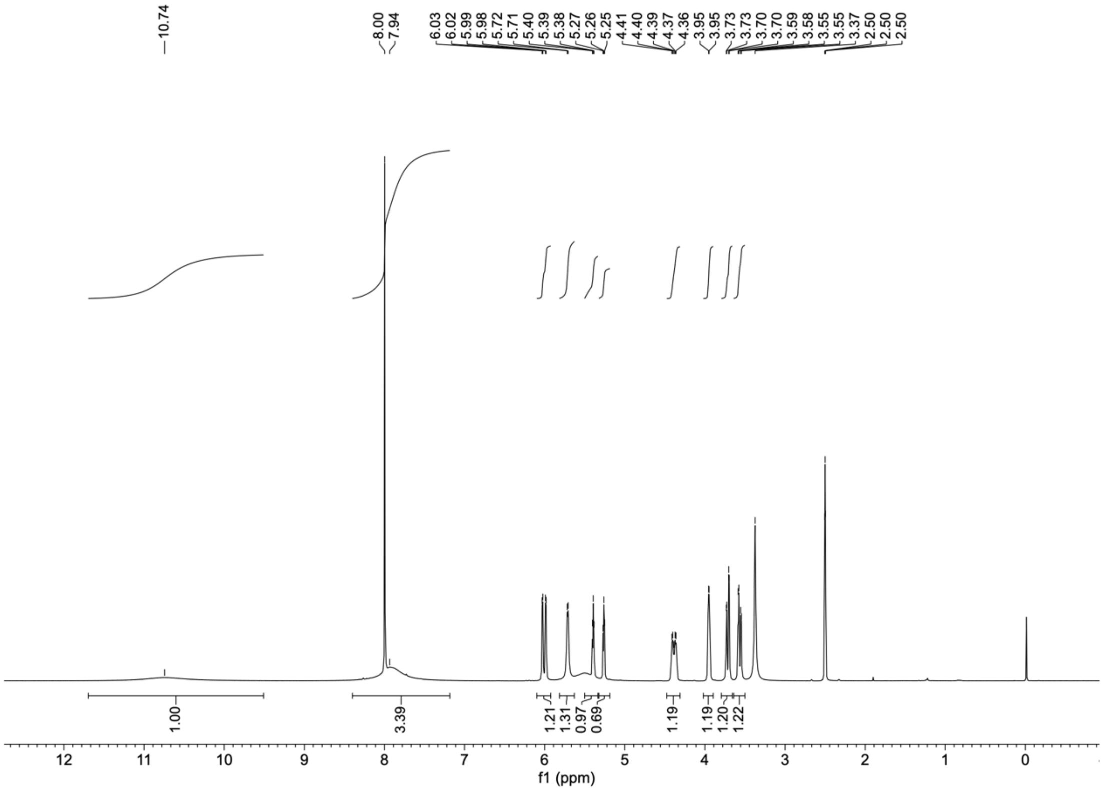

Figure S7. ^{1}H NMR spectrum of compound 2’-fluoro-isoguanosine

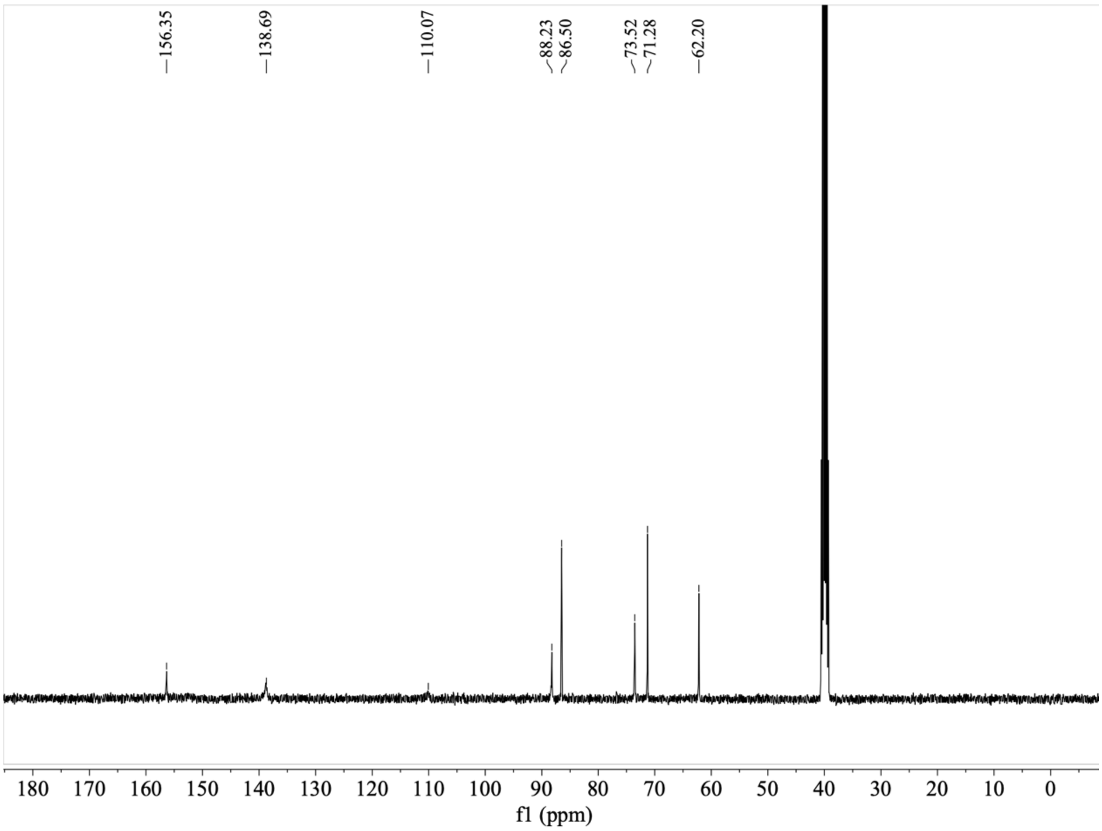

Figure S8. ^{13}C NMR spectrum of compound isoG-M

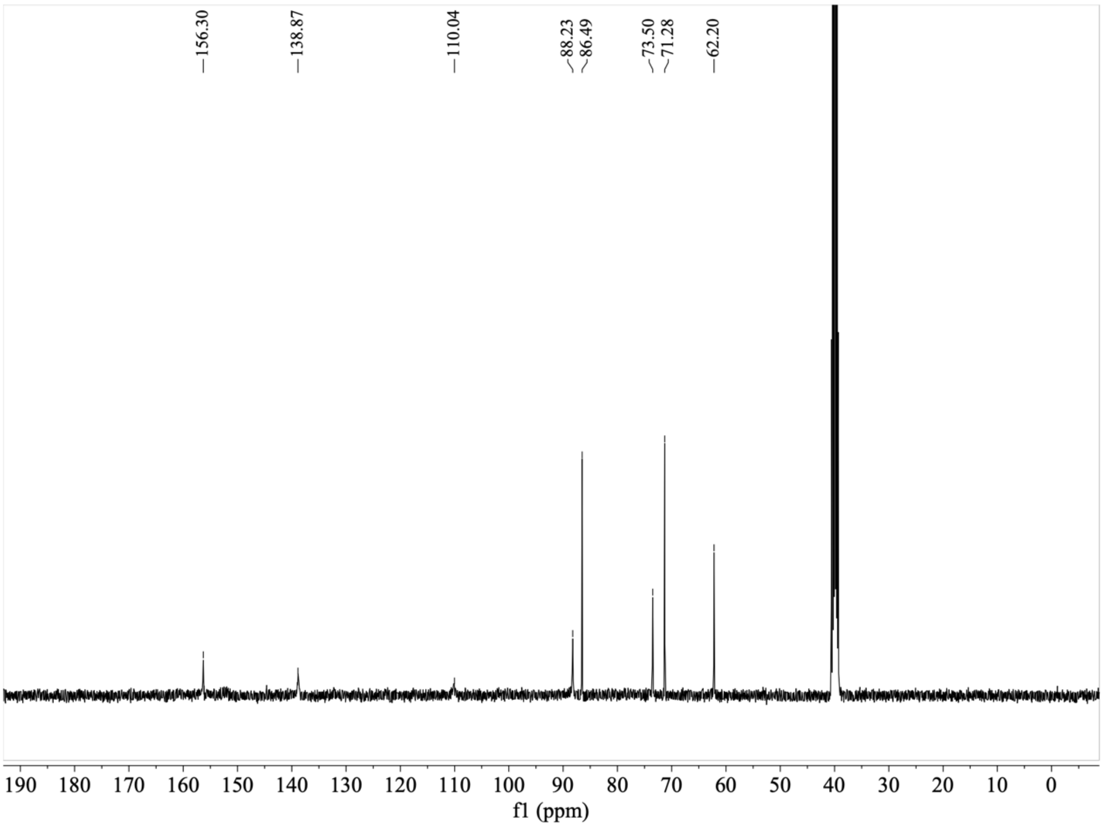

Figure S9. ^{13}C NMR spectrum of compound isoG-S

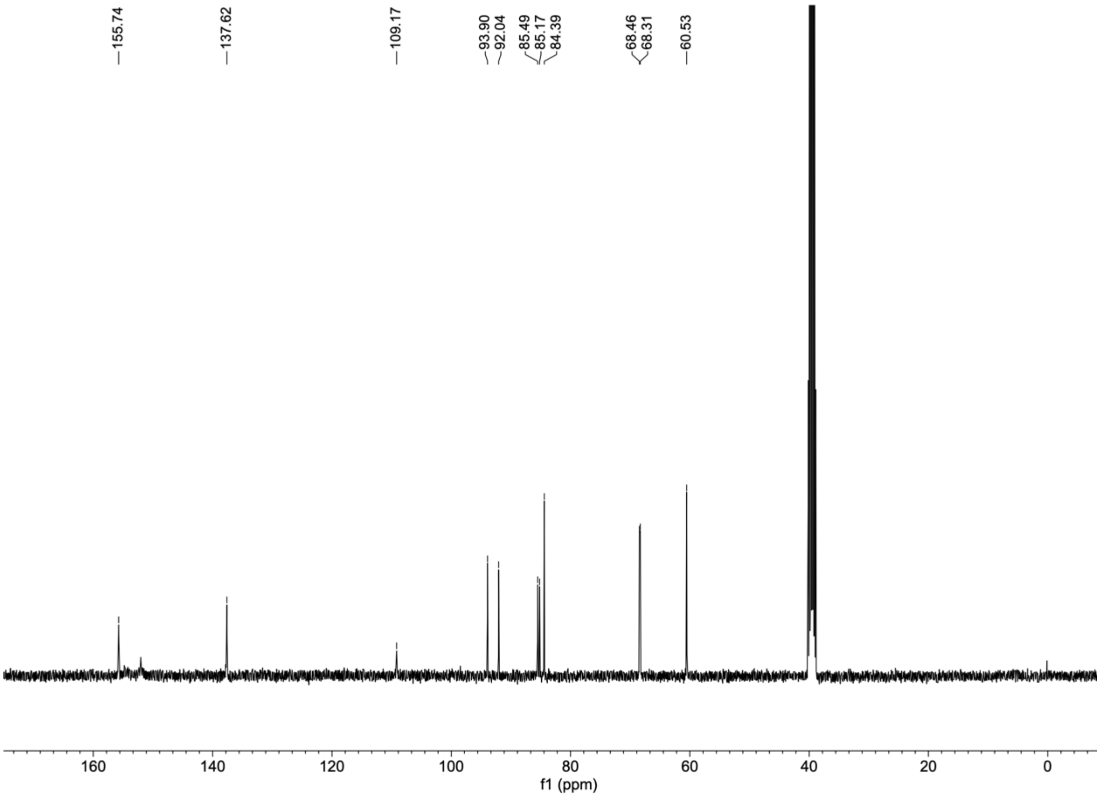

Figure S10. ^{13}C NMR spectrum of 2'-fluoro-isoguanosine

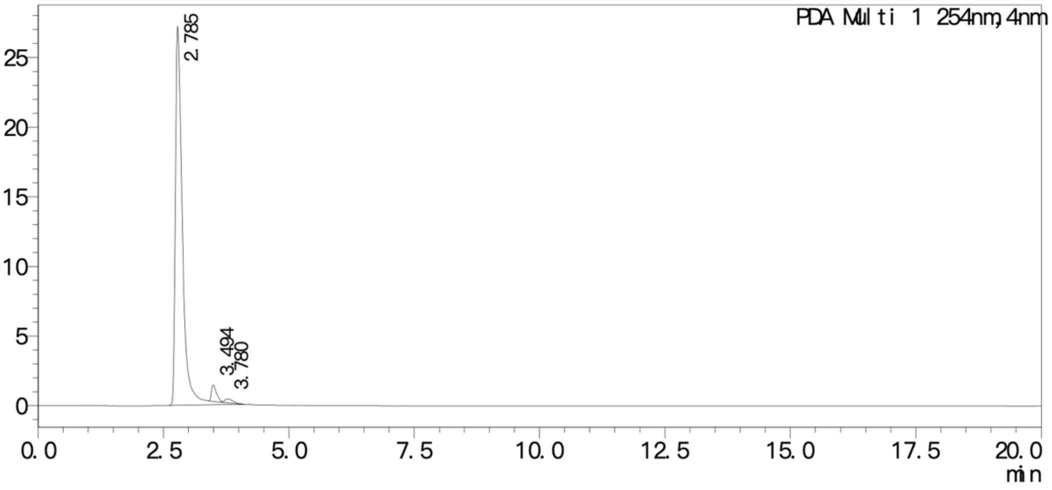

Peak number	RT[min]	Area	Height	Area%
1	2.785	268473	27212	95.97
2	3.494	8221	1184	2.94
3	3.780	3064	278	1.10
sum		279758	28675	

Figure S11. HPLC data of compound isoG-M

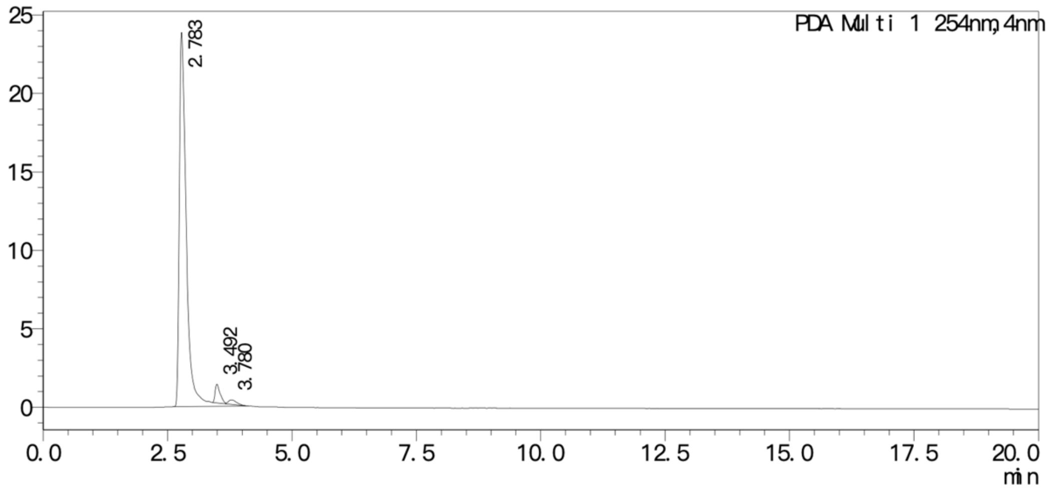

Peak number	RT[min]	Area	Height	Area%
1	2.783	237267	23853	95.39
2	3.492	8279	1192	3.33
3	3.780	3175	284	1.28
sum		248721	25328	

Figure S12. HPLC data of compound isoG-S

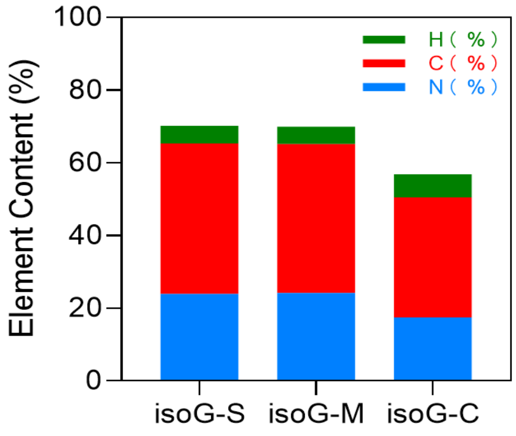

Figure S13. Element content of isoG-M,isoG-S and isoG-C.

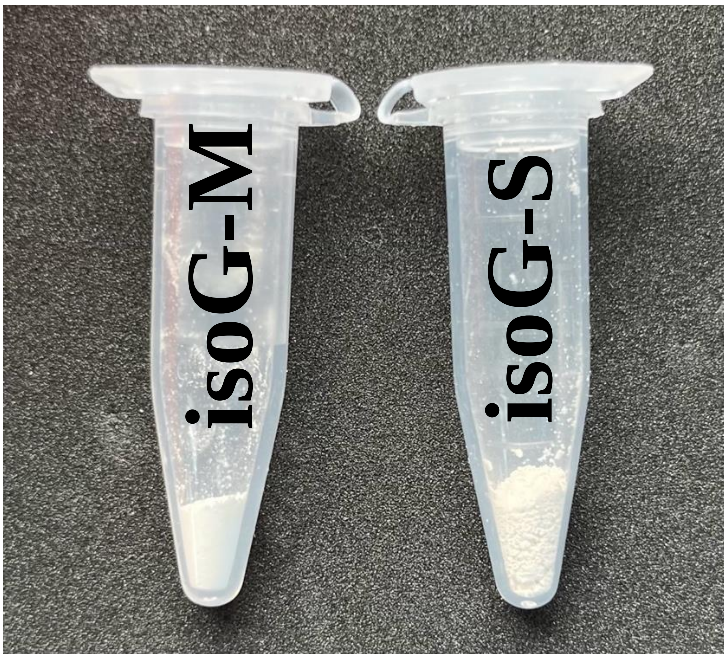

Figure S14. Powder color of isoG-M and isoG-S

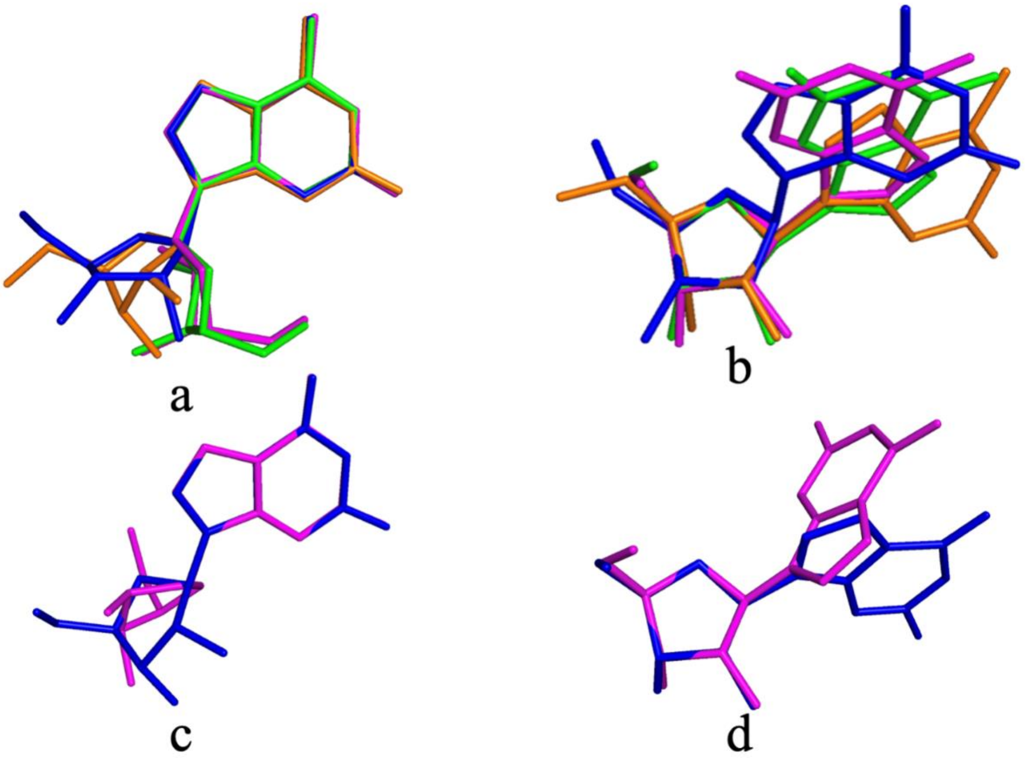

Figure S15. The molecular overlay of isoG A, B, C and D.

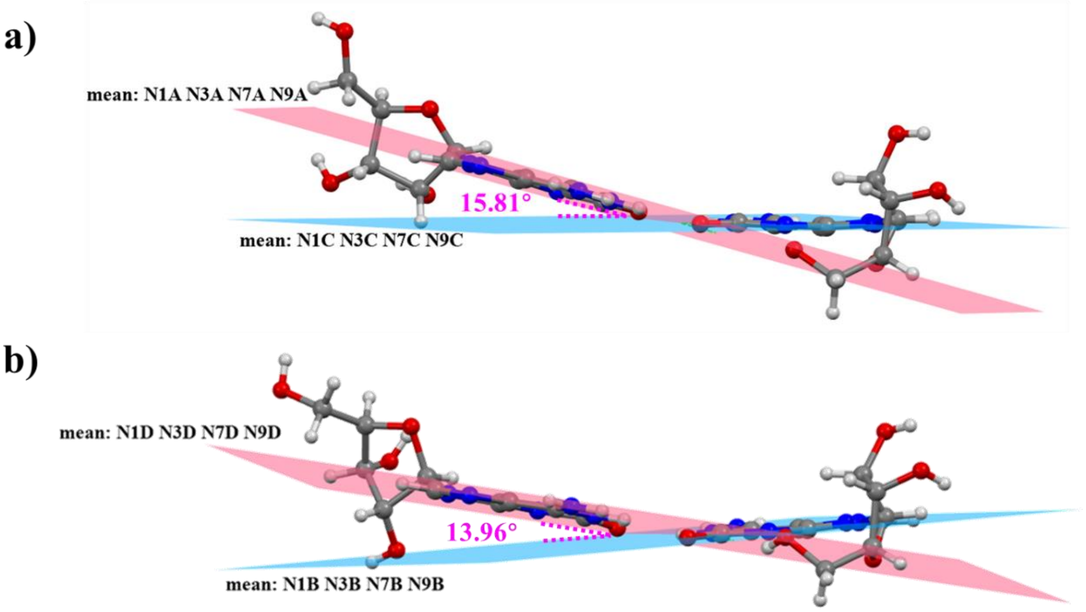

Figure S16. The bending angles of the (a) conformer A-C base pairs and (b) conformer B-D base pairs.

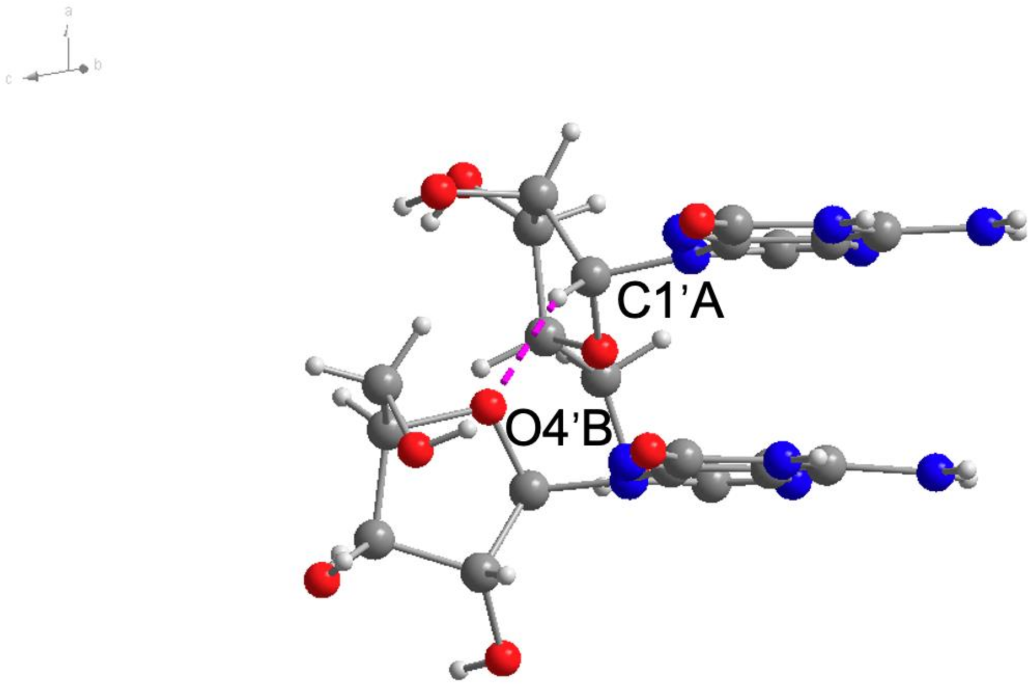

Figure S17. C1′A-H1′A···O4′B hydrogen bond.

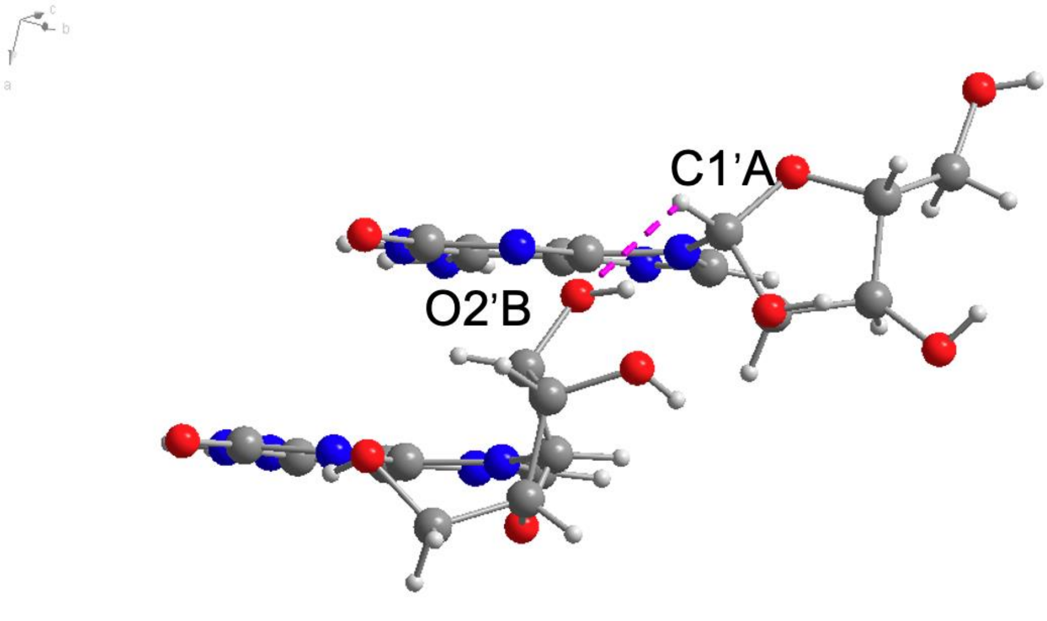

Figure S18. C1′A-H1′A···O4′B hydrogen bond.

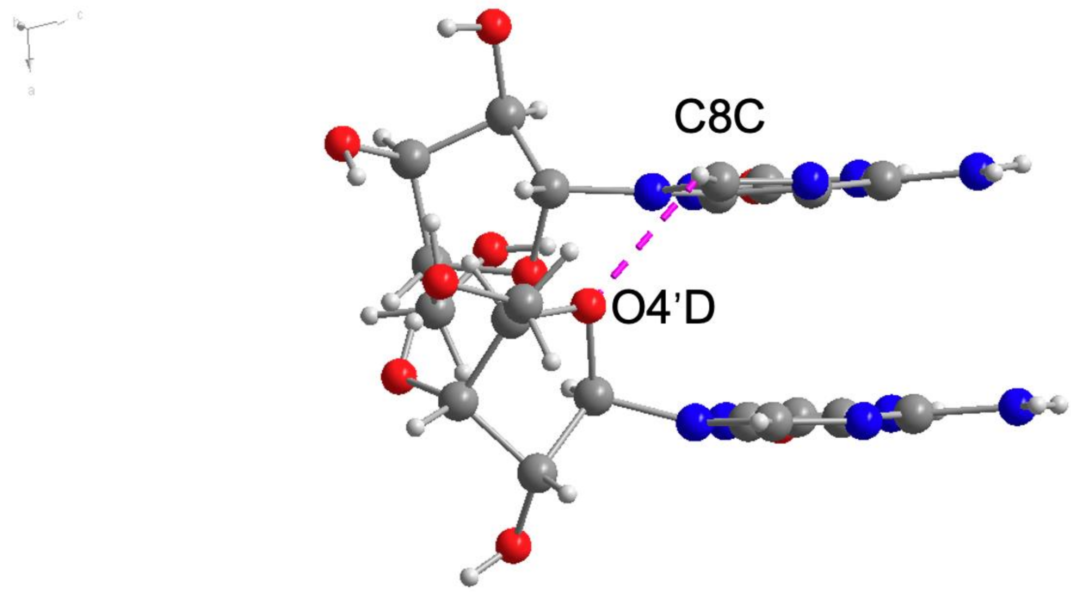

Figure S19. C8C-H8C···O4′D hydrogen bond.

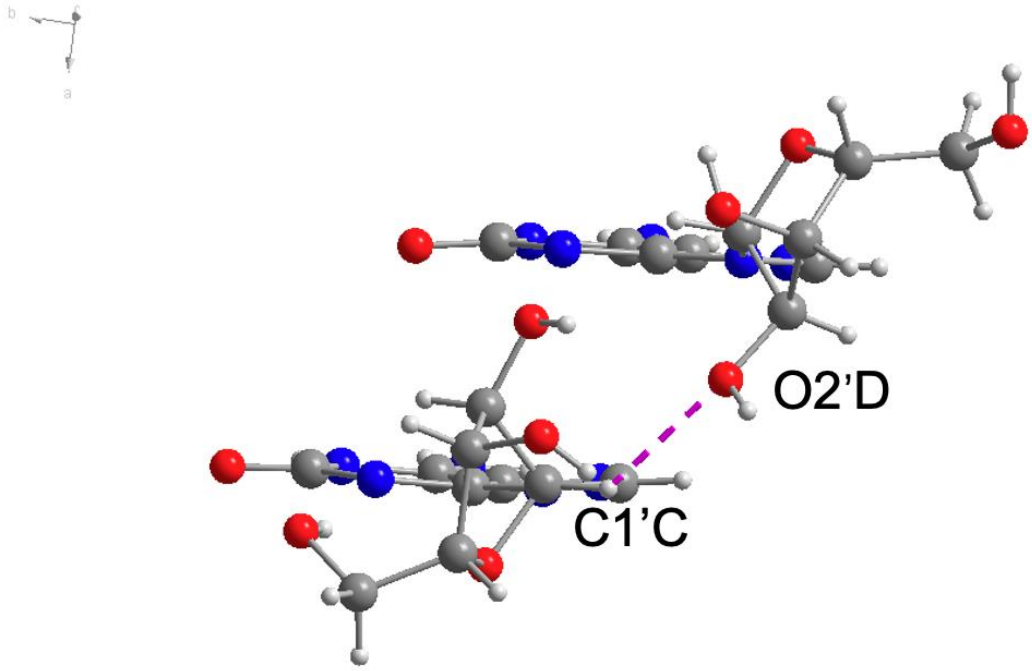

Figure S20. C1′C-H1′C···O2′D hydrogen bond.

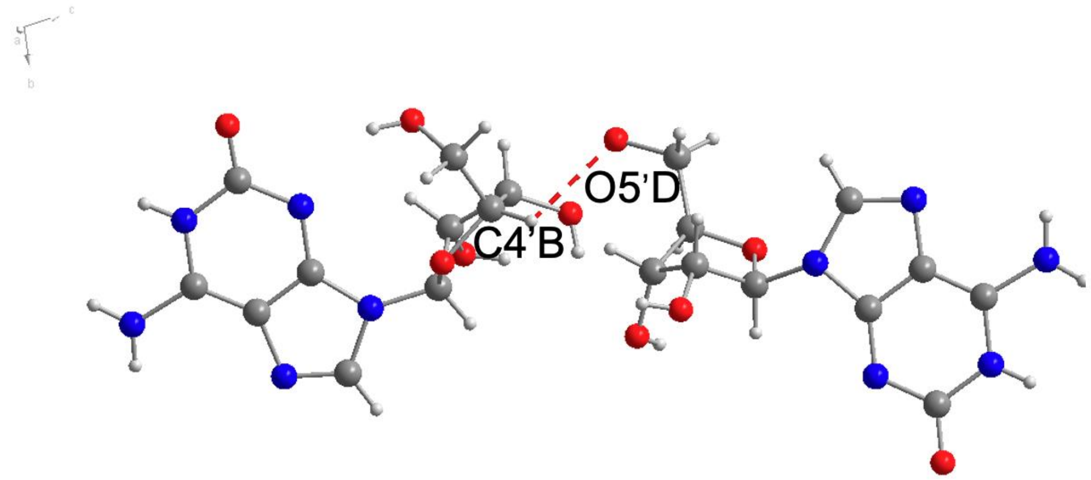

Figure S21. C4′B-H4′B⋯O5′D hydrogen bond.

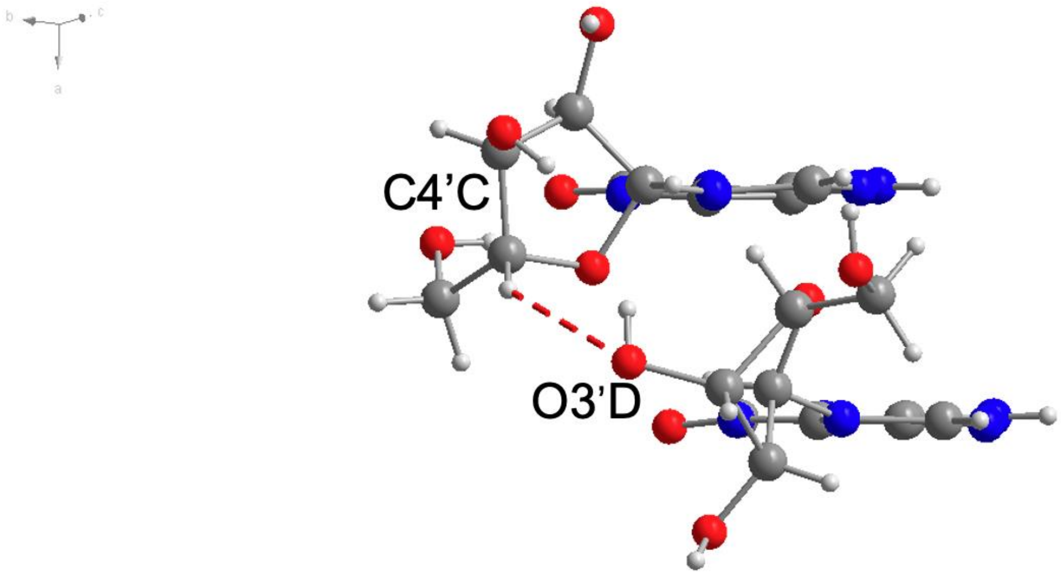

Figure S22. C4′C-H4′C⋯O3′D hydrogen bond.

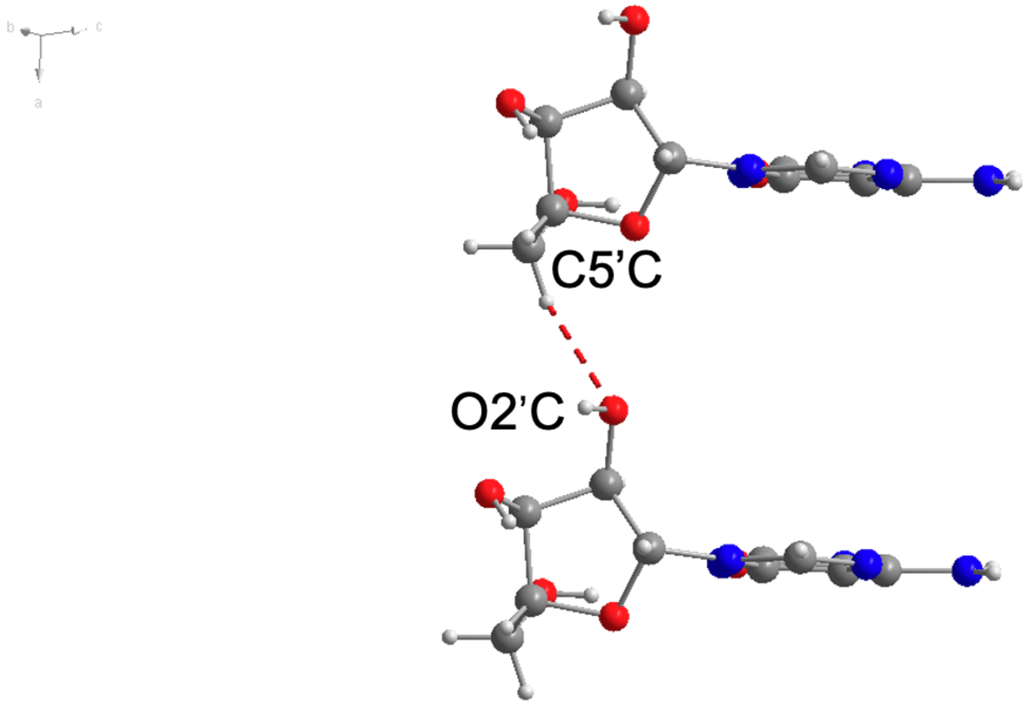

Figure S23. C5′C-H5′K··· O2′C hydrogen bond.

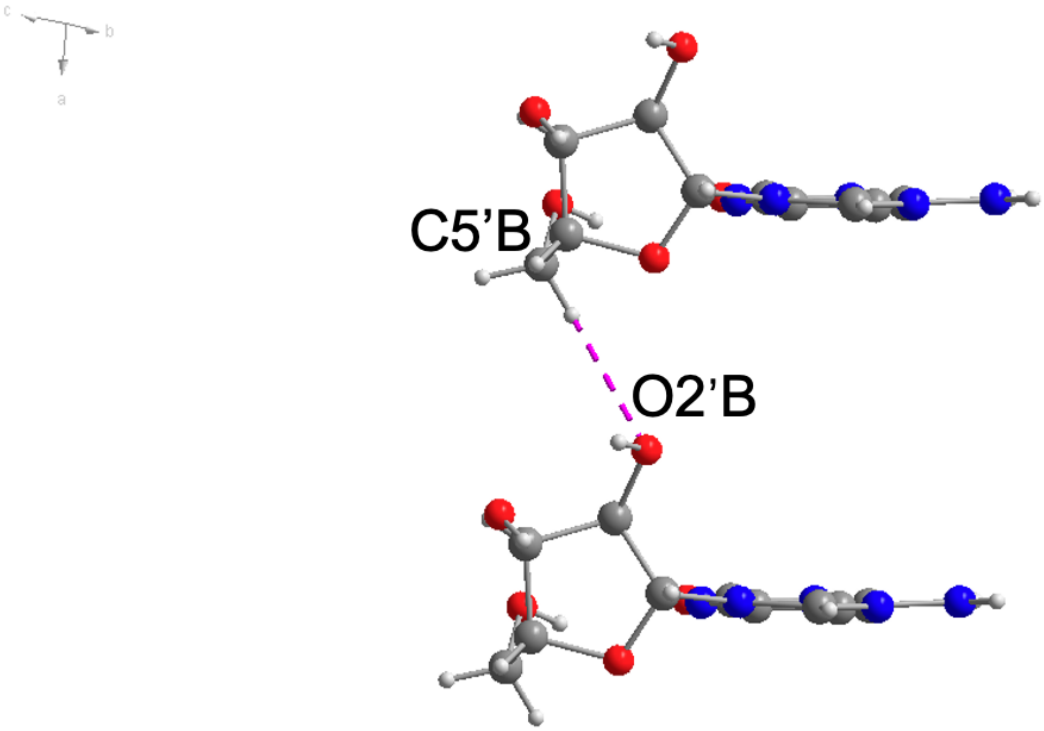

Figure S24. C5′B-H5′A··· O2′B hydrogen bond.

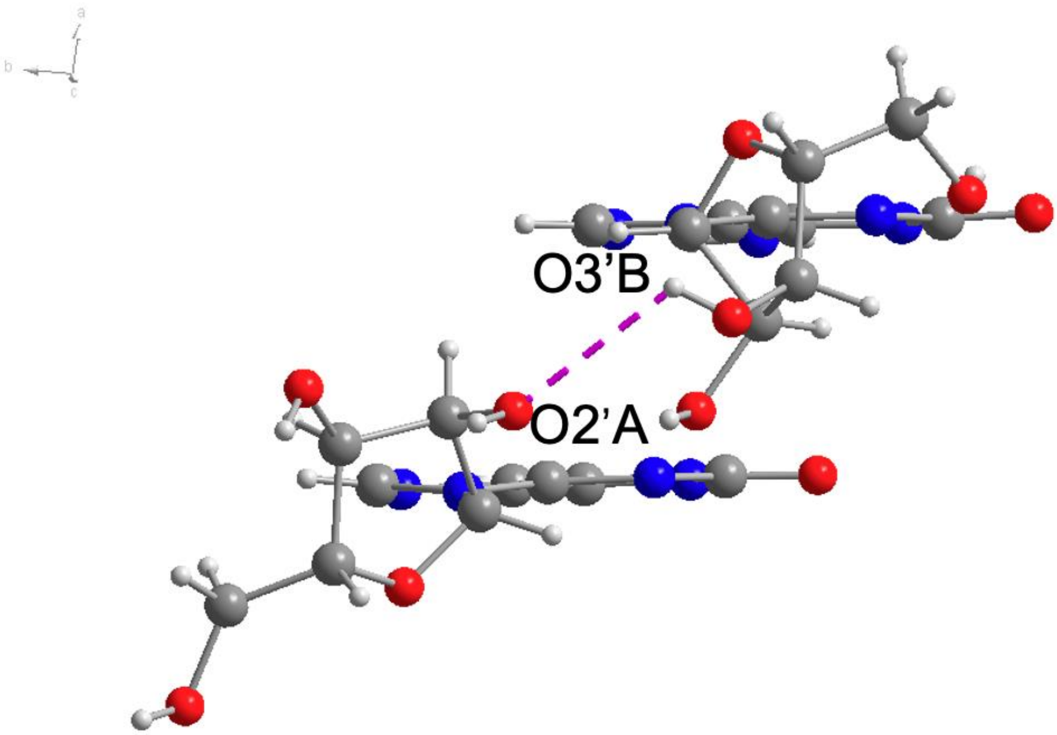

Figure S25. O3′B-H3′A··· O2′A hydrogen bond.

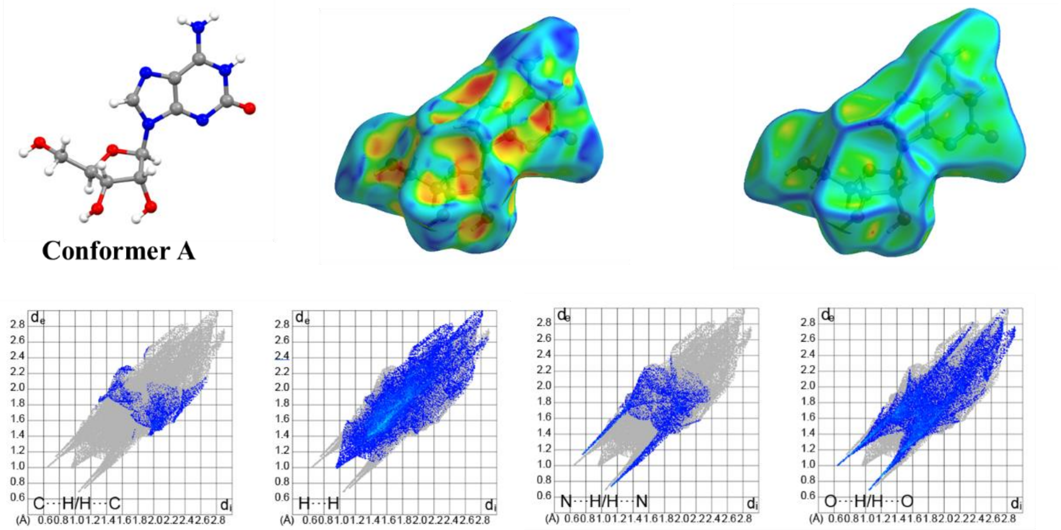

Figure S26. The Hirshfeld surfaces mapped over by d_{norm}, shape index and curvedness images of Conformer A. The classified decomposed close contacts 2D graphs of Conformer A.

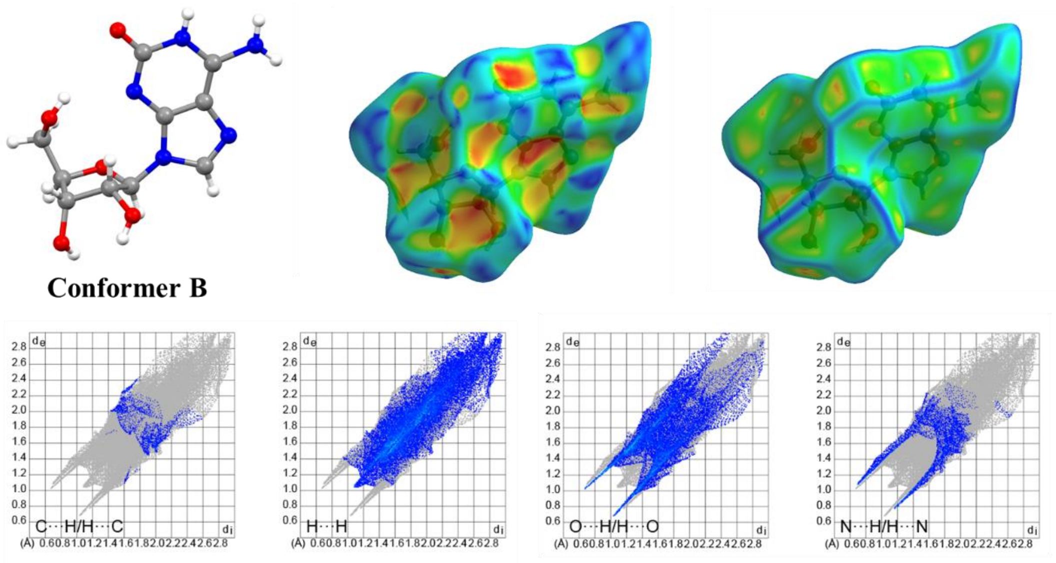

Figure S27. The Hirshfeld surfaces mapped over by d_{norm}, shape index and curvedness images of Conformer B. The classified decomposed close contacts 2D graphs of Conformer B.

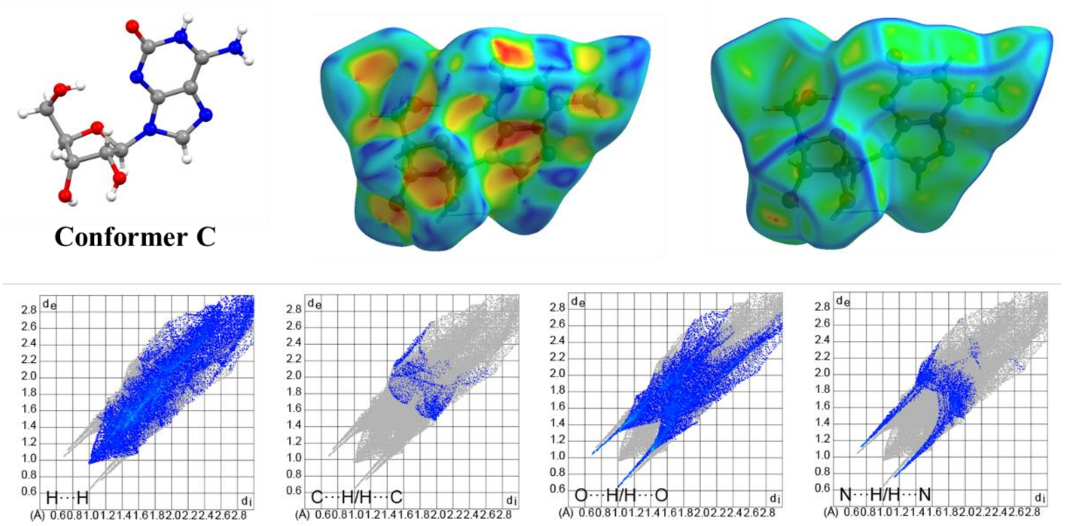

Figure S28. The Hirshfeld surfaces mapped over by d_{norm}, shape index and curvedness images of Conformer C. The classified decomposed close contacts 2D graphs of Conformer C.

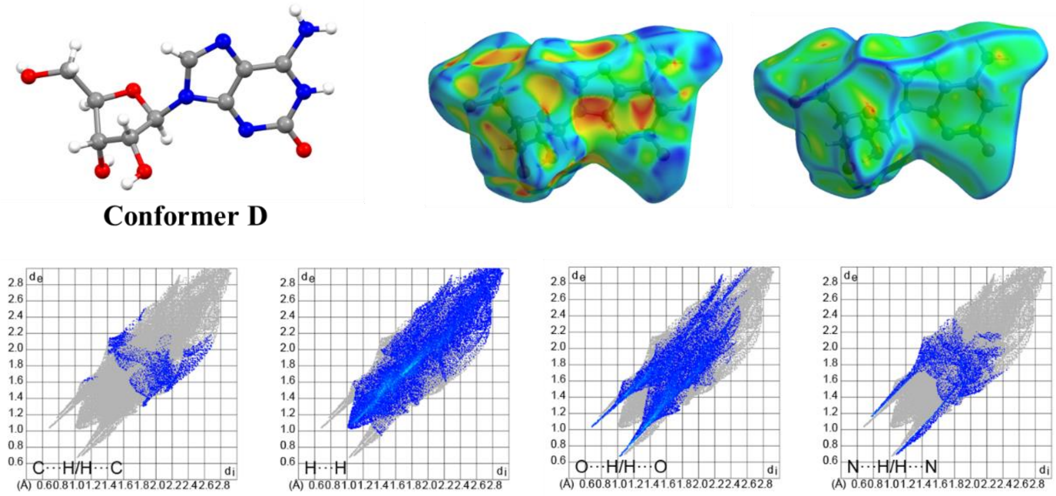

Figure S29. The Hirshfeld surfaces mapped over by d_{norm}, shape index and curvedness images of Conformer D. The classified decomposed close contacts 2D graphs of Conformer D.

Table S1. The conversion rate and yield of the product were determined by ^{1}H NMR

Compound	Conversion Rate (%)	Yield (%)
isoG	97.2	41
1	96.0	43
2	92.4	44.5

Table S2. Crystallographic data and structure refinement details of isoG.

	isoG
Empirical formula	$C_{20}H_{26}N_{10}O_{10}$
Formula weight	566.49
Crystal system	triclinic
Space group	P1
a (Å)	6.7000(13)
b (Å)	11.700(2)
c (Å)	18.000(4)
α (°)	100.30(3)
β (°)	100.00(3)
γ (°)	92.00(3)
Cell volume ($Å^3$)	1364.0(5)
Calc. density (g/cm^3)	1.379
Z	2
Temperature (K)	77.15
Data/restraints/parameters	7287/96/211
Goodness-of-fit on F^2	1.372
R_1, wR_2 [I>=2 σ (I)]	0.2152, 0.4643
R_1, wR_2 (all data)	0.2416, 0.4986
Reflections collected	15179
Independent reflections	7287 [R_{int} = 0.2819, R_{sigma} = 0.3249]
Index ranges	-8 ≤ h ≤ 8, -14 ≤ k ≤ 14, -20 ≤ l ≤ 20
2 θ range for data collection (°)	0.126-1.796
CCDC NO.	2326604

Table S3. IsoG in solid structure.

Name	isoG
Conformer Torsion angle χ (O4′-C1′-N9-C4)	A: -135.7 (7)°, *anti*<br>B: 57.6 (4)°, *syn*<br>C: 57.8 (1)°, *syn*<br>D: -111.6 (0)°, *anti*
Sugar puckering	A: C4′-exo (*P*=36.8(8)°, τm=28.3(0)°)<br>B: C2′-endo(*P*=147.1 (0)°, τm=40.6 (7)°)<br>C: C1′-exo(*P*=137.6(4)°, τm=51.7 (7)°)<br>D: C3′-exo(*P*=182.1(1)°, τm=52.4(4)°)
Torsion angle γ (O5′-C5′-C4′-C3′)	A: γ=178.0(2)°, ap (gauche, trans)<br>B: γ=30.5(2)°, +sc (gauche, gauche)<br>C: γ=46.7(8)°, ap (gauche, trans)<br>D: γ=-67.4(6)°, -sc (trans, gauche)

Table S4. G in solid structure.

Name	G
Conformer Torsion angle χ (O4′-C1′-N9-C4)	A: -137. 1(9)°, *anti*<br>B: -58.0 (7)°, *syn*
Sugar puckering	A: C1′-exo (*P*=138.3 (7)°, τm=43.3 (6)°)<br>B: C2′-endo(*P*=161.0 (1)°, τm=35.3 (1)°)
Torsion angle γ (O5′-C5′-C4′-C3′)	A: γ=46.4(4)°, ap (gauche, trans)<br>B: γ=67.9(4)°, +sc (gauche, gauche)

Table S5. The five torsion angles of the furanose rings for isoG.

Torsion angle	A	B	C	D
v0 (C4′-O4′-C1′-C2′)	-9.8(0)°	-32.4(9)°	-46.1(4)°	-17.7(8)°
v1 (O4′-C1′-C2′-C3′)	-8.9(2)°	42.7(7)°	52.1(6)°	44.0(6)°
v2 (C1′-C2′-C3′-C4′)	22.6(4)°	-34.1 (4)°	-38.2(5)°	-52.4(0)°
v3 (C2′-C3′-C4′-O4′)	-27.5(7)°	16.8(5)°	11.7(7)°	49.3(7)°
v4 (C3′-C4′-O4′-C1′)	23.8(2)°	9.5(8)°	20.8(2)°	-18.4(2)°

Table S6. The five torsion angles of the furanose rings for G

Torsion angle	A	B
v0 (C4′-O4′-C1′-C2′)	-38.5(1°)	-22.0(5)°
v1 (O4′-C1′-C2′-C3′)	44.2(4)°	34.7(4)°
v2 (C1′-C2′-C3′-C4′)	-32.4(1)°	-33.3 (9)°
v3 (C2′-C3′-C4′-O4′)	11.0(8)°	21.6(0)°
v4 (C3′-C4′-O4′-C1′)	16.9(7)°	0.1(7)°

Table S7. The hydrogen-bond geometry for isoG (Å, °)

D-H-A	d(D-H)	d(H..A)	<DHA	d(D..A)
O2'A-H2'F···O3'A	0.84	2.268	116.26	2.745
O5'B-H5'B···N3B	0.84	2.346	130.78	2.963
C2'B-H2'B···N3B	1	2.615	129.76	3.345
O2'B-H2'A···O3'B	0.84	2.286	116.97	2.77
C2'C-H2'G···N3C	1	2.588	127.4	3.293
C2'C-H2'G···O5'C	1	2.649	112.12	3.165
O2'C-H2'H···O3'C	0.84	2.213	118.85	2.719
O5'C-H5'J···N3C	0.84	2.313	163.44	3.127
N1B-H1B···O2D	0.88	1.828	167.45	2.693
N1D-H1D···O2B	0.88	1.84	167.34	2.706
N6B-H6BA···N7D [x, y+1, z]	0.88	1.903	169.65	2.773
N6D-H6DA···N7B [x, y-1, z]	0.88	2.061	161.55	2.909
N6A-H6AB···N7C [x+1, y+1, z]	0.88	2.016	161.55	2.864
N6C-H6CB···N7A [x-1, y-1, z]	0.88	1.776	170.96	2.649
N1A-H1A···O2C [x+1, y, z]	0.88	1.969	158.26	2.806
N1C-H1C···O2A [x-1, y, z]	0.88	1.858	168.54	2.726
C1'A-H1'A···O4'B	1	2.26	154.14	3.19
O2'B-H2'A···O2'A [x-1, y, z]	0.84	2.162	135.49	2.823
O2'C-H2'H···O2'D [x-1, y, z]	0.84	2.428	116.26	2.899
C1'C-H1'C···O4'D	1	2.653	123.2	3.308
O2'D-H2'C···O3'B [x, y-1, z-1]	0.84	2.722	152.12	3.487
O5'D-H5'F···O3'B [x-1, y-1, z-1]	0.84	2.399	129.62	3.005
O2'A-H2'F···O3'D [x+1, y+1, z+1]	0.84	2.038	132.24	2.676
C8B-H8B···O4'A	0.95	2.689	118.03	3.246
C1'A-H1'A···O2'B [x+1, y, z]	1	2.449	120.75	3.082
C8C-H8C···O4'D	0.95	2.587	117.06	3.136
C1'C-H1'C···O2'D [x-1, y, z]	1	2.38	135.39	3.17
C4'B-H4'B···O5'D [x+1, y+1, z+1]	1	2.406	113.35	2.948
C4'C-H4'C···O3'D	1	2.609	138.76	3.425
C5'C-H5'K···O2'C [x+1, y, z]	0.99	2.571	143.63	3.419
C5'B-H5'A···O2'B [x+1, y, z]	0.99	2.73	144.79	3.584
C1'D-H1'D···O2'C [x+1, y, z]	1	2.564	136.38	3.36
C1'D-H1'D···O4'C	1	2.56	147.09	3.443
O3'B-H3'A···O2'A [x-1, y, z]	0.84	2.728	114.23	3.167
